# Weakly nonlinear analysis on synchronization and oscillation quenching of coupled mechanical oscillators

**DOI:** 10.1038/s41598-024-51843-9

**Published:** 2024-01-17

**Authors:** Yusuke Kato, Hiroshi Kori

**Affiliations:** Department of Complexity Science and Engineering, Graduate School of Frontier Sciences, The University of Tokyo, Kashiwa, Chiba 277-8561 Japan

**Keywords:** Nonlinear phenomena, Applied mathematics

## Abstract

Various oscillatory phenomena occur in the world. Because some are associated with abnormal states (e.g. epilepsy), it is important to establish ways to terminate oscillations by external stimuli. However, despite the prior development of techniques for stabilizing unstable oscillations, relatively few studies address the transition from oscillatory to resting state in nonlinear dynamics. This study mainly analyzes the oscillation-quenching of metronomes on a platform as an example of such transitions. To facilitate the analysis, we describe the impulsive force (escapement mechanism) of a metronome by a fifth-order polynomial. By performing both averaging approximation and numerical simulation, we obtain a phase diagram for synchronization and oscillation quenching. We find that quenching occurs when the feedback to the oscillator increases, which will help explore the general principle regarding the state transition from oscillatory to resting state. We also numerically investigate the bifurcation of out-of-phase synchronization and beat-like solution. Despite the simplicity, our model successfully reproduces essential phenomena in interacting mechanical clocks, such as the bistability of in-phase and anti-phase synchrony and oscillation quenching occurring for a large mass ratio between the oscillator and the platform. We believe that our simple model will contribute to future analyses of other dynamics of mechanical clocks.

## Introduction

Oscillatory phenomena are widely observed in nature and society. There are both desirable and undesirable rhythms. For example, some rhythms in the human body, such as the heartbeat and circadian clock, are essential for homeostasis, while others appear with diseases, such as abnormal oscillations of neuronal action potentials in Parkinson’s disease^1^ or epilepsy^2,3^. Thus, it is expected that stabilizing the necessary oscillations and removing the abnormal oscillations will contribute to healthy biological rhythms and the treatment of diseases. Mathematically, an oscillatory state corresponds to a limit cycle of a dynamical system^4^. Therefore, developing techniques to stabilize or destabilize limit cycles by external perturbations is important.

Several methods for stabilizing unstable limit cycles were established in the 1990s^5,6^. On the other hand, regarding the methods to destabilize the stable limit cycles, although various state-transition methods between multiple limit cycles have been extensively studied^7–11^, few studies have explored the general principle for state transition from stable limit cycles to stable fixed points^12–15^. Establishing methods for transferring the system state from a stable limit cycle to a stable fixed point is crucial for annihilating undesirable rhythms^14,15^.

In the present paper, we consider the metronome as an example of a bistable system with a stable limit cycle and stable fixed point. We focus on the oscillation-stopping phenomenon (oscillation quenching) of the metronome on a platform, in which the metronome stops vibrating after oscillating for a while. Below, we explain the details of our study referring to the previous studies on metronome dynamics.

## Related works on the dynamics of mechanical oscillators

A metronome is a mechanical device in which a needle with a weight continuously oscillates. Its characteristic structure is that the combination of a spring and gear provides torque in the same direction as the motion of the needle when the needle reaches a certain position^16^. This structure, called an escapement mechanism, enables the metronome to oscillate by counteracting the damping force caused by friction. To investigate the various dynamical behaviors of metronomes and their mechanical analogues, e.g. pendulum clocks, numerous experimental studies have been performed^17–27^. One of the most famous dynamics of coupled metronomes is synchronization, in which the timing of the metronome oscillation is aligned when multiple metronomes are placed on a common platform. Synchronization was first discovered in coupled pendulum clocks by Huygens^18^, and many experiments have since been conducted in various settings^17,19–21,25–27^. In particular, it is known that both in-phase and anti-phase synchronizations, or only one of them, can be observed depending on the experimental situation^21^. Another unique behavior is oscillation quenching, which has been observed in metronomes on a movable platform^27^ or pendulum clocks suspended on a movable board^18^. However, although a lot of previous studies focused on the synchronization of metronomes, few investigated the oscillation quenching.

Numerous modeling studies have also been performed to analyze the dynamics of metronomes and pendulum clocks^16–31^. As summarized in Ref^31^, several challenges exist with these studies. The first is the modeling of the escapement mechanism. To describe the escapement mechanism, it is considered appropriate to use functions that provide torque in the same direction as pendulum motion. In the previous studies, the van der Pol-type function^17,22,29^, the piecewise linear function^19,20,23,24,26,30^, Dirac’s delta function^27,31^, and the function that instantaneously changes the angular velocity^18^ or the kinetic energy^25^ of the pendulum at specific positions have been used. Another challenge is that the motion equation of a metronome generally becomes a nonlinear ordinary differential equation (ODE) that cannot be solved explicitly. To analyze the nonlinear motion equation, an averaging approximation^17,27,31^ and Poincaré map^18,25^ have been applied.

Recently, Goldsztein et al. analyzed a mathematical model of two metronomes on a movable platform and obtained a phase diagram for in-phase and anti-phase synchronization^31^. In particular, they succeeded in explaining some of the past experiments by considering the metronome’s nonlinearity caused by its pendulum structure^31^; that is, they expanded the usual linear small-angle approximation ($\sin \theta \simeq \theta$) to include the nonlinear term ($\sin \theta \simeq \theta + c \theta ^3$ with sufficiently small *c*). Seeking a better agreement with the experimental results, they also created a more realistic model by assuming Coulomb friction as the damping of the platform^27^. Although these studies are elaborate and sophisticated in terms of both modeling methods and analytical techniques, there are several open questions. In their first study^31^, the analytical method (i.e. averaging approximation) was applied only when the amplitude of the metronome was larger than a certain threshold value, preventing the analysis of oscillation quenching. In the second study^27^, although this issue was resolved (i.e. the averaged system was valid even when the amplitude was small), a stability analysis was not performed because the averaged system contained discontinuous functions. The behavior of the equation of motion was tested only by numerical simulations, and a phase diagram was not created.

## Study purpose and structure of this paper

We are particularly concerned with oscillation quenching because this phenomenon can be considered as an example of a state transition from a stable limit cycle (oscillating state) to a stable fixed point (resting state) when the oscillators receive the feedback resulting from their motion via the platform. Thus, this study aims to treat both the synchronization and oscillation quenching in a unified manner, that is, using the same mathematical model. One of the reasons why the analysis of oscillation quenching was difficult in the previous studies^27,31^ is that the authors attempted to make the model realistic by describing the escapement mechanism with a delta function and considering both the nonlinearity of the pendulum structure and the Coulomb friction acting on the platform. Thus, to facilitate the analysis, we model the metronome as a linear spring pendulum, neglect the damping of the platform, and simulate the escapement with several smooth functions, particularly a polynomial of order five. Owing to these simplifications, we analytically and numerically obtain a phase diagram for both synchronization and oscillation quenching, which has not been obtained in the previous studies on metronomes.

The remainder of this paper is organized as follows. First, we consider the case of a single metronome on a movable platform. Assuming that both the escapement mechanism and damping force are sufficiently small, we treat the entire system as a weakly nonlinear oscillator and analyze the equation of motion using an averaging approximation. We use several functions to represent the escapement mechanism and confirm that the averaged system reproduces the bistability and oscillation quenching of a real metronome. We then expand our model to the case of two identical metronomes on a movable platform, where we adopt a fifth-order polynomial as the escapement mechanism to make the analysis easier. Assuming that the mass ratio of the metronome to the platform is sufficiently small, we perform an averaging approximation and a linear stability analysis to obtain a phase diagram for the in-phase synchronization, anti-phase synchronization, and oscillation quenching. We verify the analysis by numerically integrating the equations of motion before the averaging approximation and plotting the results on the same phase diagram. Finally, we provide a summary and discussion.

## One metronome

### Model

**Figure 1:**
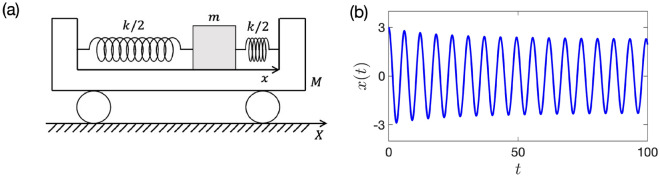
(**a**) The model of one metronome on a movable platform. To facilitate the later analysis, we ignore the pendulum structure of the real metronome. (**b**) Typical dynamics of the nondimensional motion Eq. (6). We set *g* as Eq. (24), ${\varepsilon }= 0.01$, $a=4$, $b=1$, $\alpha = 0.5$, and $(x(0),\dot{x}(0)) = (3,0)$.

Figure 1a illustrates our model of one metronome on a movable platform. In this model, a point mass *m* is connected by two springs with spring constant *k*/2 to a platform of mass *M*. The platform has one degree of freedom and moves freely. The variables *X*(*t*) and *x*(*t*) are the positions of the platform relative to the floor and the mass relative to the platform, respectively.

We consider the following two forces that act from the platform to the mass point: the damping force proportional to the velocity of the mass, $\dot{x} {:}{=}dx/dt$, and the driving force describing the escapement mechanism given as a function of *x* and $\dot{x}$. By neglecting the mass of the springs, the air resistance of the mass and platform, and the damping of the platform due to friction with the floor, we obtain the following motion equation:

1 $$\begin{aligned} m\ddot{x} + kx + \gamma \dot{x} - \delta f(x,{\dot{x}}) + m{\ddot{X}} = 0, \end{aligned}$$

where $\gamma$ is the damping coefficient, $\delta$ represents the magnitude of the escapement mechanism, and $f(x,{\dot{x}})$ is a dimensionless function that shows the nature of the escapement mechanism.

We denote the position of the center of mass as $x_\text{c}$. Then,

2 $$\begin{aligned} x_\text{c} = \frac{MX + m(X+x)}{M+m}. \end{aligned}$$

Since we assume that there is no external force acting on the entire system, the equation $\frac{d^2 x_\text{c}}{d t^2} = 0$ follows. Thus,

3 $$\begin{aligned} {\ddot{X}} = -\frac{m}{M+m}\ddot{x}. \end{aligned}$$

Substituting Eq. (3) into Eq. (1), we have

4 $$\begin{aligned} \frac{Mm}{M+m}\ddot{x} + kx + \gamma \dot{x} - \delta f(x,{\dot{x}}) = 0. \end{aligned}$$

We then nondimensionalize Eq. (4). By introducing a small dimensionless parameter ${\varepsilon }$ and the following quantities:

5 $$\begin{aligned} \mu {:}{=}\frac{m}{M},\quad \omega {:}{=}\sqrt{\frac{(1 + \mu )k}{m}}, \quad \tau {:}{=}\omega t, \quad \alpha {:}{=}\frac{\omega \gamma }{{\varepsilon }k}, \quad {{\hat{x}}} {:}{=}\frac{k x}{\delta }, \quad g \left( {{\hat{x}}}, \frac{d {{\hat{x}}}}{d \tau } \right) {:}{=}\frac{1}{{\varepsilon }}f \left( \frac{\delta {{\hat{x}}}}{k}, \frac{\omega \delta }{k}\frac{d {{\hat{x}}}}{d \tau }\right) . \end{aligned}$$

and renaming $\tau \rightarrow t$ and ${{\hat{x}}} \rightarrow x$, we transform Eq. (4) into the following dimensionless system:

6 $$\begin{aligned} \ddot{x} + x = {\varepsilon }\left( - \alpha \dot{x} + g(x,{\dot{x}}) \right) . \end{aligned}$$

Here, we assume $x = O(1)$, $\dot{x} = O(1)$, $\alpha = O(1)$ and $g(x,\dot{x}) = O(1)$. We show the typical dynamics of Eq. (6) in Fig. 1b.

### Analysis

We perform an averaging approximation to the system (6). We rewrite Eq. (6) as

7a $$\begin{aligned} \dot{x}&= y, \end{aligned}$$

7b $$\begin{aligned} \dot{y}&= -x + {\varepsilon }\left( - \alpha y + g(x,y) \right) . \end{aligned}$$

We transform the variables *x*(*t*) and *y*(*t*) into new variables *r*(*t*), $\theta (t)$, and $\phi (t)$ that satisfy $r_i(t) \ge 0$ and the following equations:

8a $$\begin{aligned} x(t)&= r(t) \cos (t + \theta (t)) = r(t) \cos \phi (t), \end{aligned}$$

8b $$\begin{aligned} y(t)&= - r(t) \sin (t + \theta (t)) = - r(t) \sin \phi (t), \end{aligned}$$

where

9 $$\begin{aligned} \phi (t) {:}{=}t + \theta (t). \end{aligned}$$

Then, Eq. (7) is transformed into

10a $$\begin{aligned} \dot{r}&= - {\varepsilon }\sin \phi \left( \alpha r \sin \phi + g(r \cos \phi , -r \sin \phi ) \right) , \end{aligned}$$

10b $$\begin{aligned} {{\dot{\theta }}}&= - \frac{{\varepsilon }}{r} \cos \phi \left( \alpha r \sin \phi + g(r \cos \phi , -r \sin \phi ) \right) . \end{aligned}$$

See Supplementary Information for the derivation of Eq. (10).

Since the estimate $r(t) = O(1)$ follows from the assumptions $x(t) = O(1)$ and $y(t) = O(1)$, Eq. (10) suggests that the time-scale of *r*(*t*) and $\theta (t)$ are $O({\varepsilon }^{-1})$ and thus much larger than the time-scale of metronome’s oscillation period $2\pi$ (i.e. the time-scale of *x*(*t*) in Eq. (8a)). Therefore, we can safely replace $\dot{r}$ and ${{\dot{\theta }}}$ with their time average over $2 \pi$. Namely, we approximate the right-hand sides of Eq. (10) with their time average as below:

11a $$\begin{aligned} \dot{r}&\simeq - \frac{{\varepsilon }}{2\pi } \int _0^{2\pi } dt \sin \phi \left( \alpha r \sin \phi + g(r \cos \phi , -r \sin \phi ) \right) \nonumber \\&= - \frac{{\varepsilon }}{2\pi } \int _0^{2\pi } d\phi \sin \phi \left( \alpha r \sin \phi + g(r \cos \phi , -r \sin \phi ) \right) = -{\varepsilon }\left( \frac{\alpha r}{2} + {{\bar{g}}}_1(r) \right) , \end{aligned}$$

11b $$\begin{aligned} {{\dot{\theta }}}&\simeq - \frac{{\varepsilon }}{2 \pi r} \int _0^{2\pi } dt \cos \phi \left( \alpha r \sin \phi + g(r \cos \phi , -r \sin \phi ) \right) \nonumber \\&= - \frac{{\varepsilon }}{2 \pi r} \int _0^{2\pi } d\phi \cos \phi \left( \alpha r \sin \phi + g(r \cos \phi , -r \sin \phi ) \right) = - \frac{{\varepsilon }}{r} {{\bar{g}}}_2(r), \end{aligned}$$

where

12a $$\begin{aligned} {{\bar{g}}}_1(r)&= \frac{1}{2 \pi } \int _0^{2\pi } d\phi \ g(r \cos \phi , -r \sin \phi ) \sin \phi , \end{aligned}$$

12b $$\begin{aligned} {{\bar{g}}}_2(r)&= \frac{1}{2 \pi } \int _0^{2\pi } d\phi \ g(r \cos \phi , -r \sin \phi ) \cos \phi . \end{aligned}$$

Note that we regard *r* and $\theta$ as constants when we calculate the integrals in Eq. (11): this is because the change of these variables during the integral interval $[0, 2\pi ]$ is $O({\varepsilon })$ and thus can be negligible in the lowest-order approximation. This approximation method is widely known as averaging method^32,33^ (or Krylov-Bogoliubov averaging method^34^), which is mathematically justified by near identity transformation^35,36^. Hereafter, we replace the approximately equal sign ($\simeq$) in Eq. (11) with the equal sign ($=$).

In what follows, we consider concrete functions as *g*(*x*, *y*) and obtain ${{\bar{g}}}_{i}(r)\; (i=1,2)$ given by Eq. (12).

#### Features of escapement mechanism

We first discuss appropriate functions to model the escapement mechanism. Considering a real metronome, it is natural to assume that *g*(*x*, *y*) takes non-zero values only when *x* and *y* have the same sign. This is because the escapement mechanism of a real metronome works when the pendulum position is the right from the center and moves to the right, or the pendulum position is the left from the center and moves to the left. We set *g*(*x*, *y*) to match this assumption.

Below, we describe the three models where *g*(*x*, *y*) is a piecewise linear function, a rational function with a numerator of degree 3 and a denominator of degree 4, and a 5th-order polynomial. In Supplementary Information, we describe another model where *g*(*x*, *y*) is a rational function with a linear numerator and a quadratic denominator.

#### Model (i)

**Figure 2:**
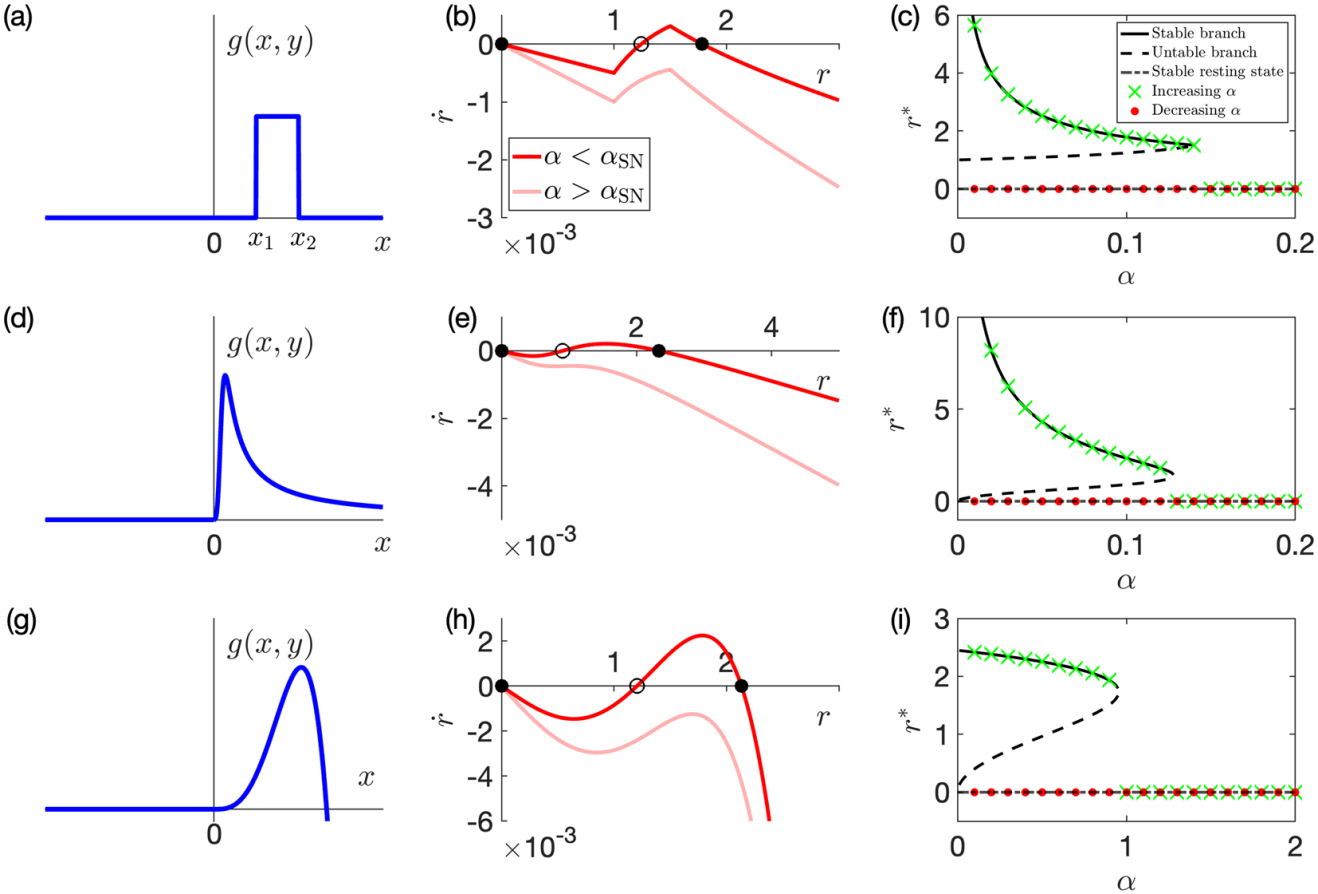
Left column: the shape of *g*(*x*, *y*) with positive *y* in Model (i) (Panel (**a**)), (ii) (Panel (**d**)), and (iii) (Panel (**g**)). Middle column: the flow for *r* dynamics in Model (i) (Panel (**b**)), Model (ii) (Panel (**e**)), and Model (iii) (Panel (**h**)). The black dots and black circles in each panel represent the stable and unstable fixed points, respectively. The parameters are as follows: ${\varepsilon }= 0.01$ for all three panels, $x_1 = 1.0,\, x_2 = 1.5,\, \alpha = 0.1$ and 0.2, for panel (**b**), $\alpha = 0.1$ and 0.2 for panel (**e**), $a=4,\, b=1,\, \alpha = 0.7$ and 1.1 for panel (**h**). Right column: the bifurcation diagram for *r* obtained by both the averaging approximation and the numerical simulation of Eq. (6) where *g* is given by Model (i) (Panel (**c**)), Model (ii) (Panel (**f**)), and Model (iii) (Panel (**i**)). The solid, dashed, and dash-dotted lines correspond to the analytically obtained stable fixed point ($r^*=r_{\text{stable}}$), the unstable fixed point ($r^* = r_{\text{unstable}}$), and the trivial fixed point ($r^* = 0$), respectively. The green cross marks show the numerically obtained fixed points in the case where $\alpha$ increases, while the red dots show those in the case where $\alpha$ decreases. For simulation, we first use $\alpha = \alpha _{\text{min}}$ and then increase $\alpha$ by $\alpha _{\text{inc}}$ until $\alpha = \alpha _{\text{max}}$. Next, we decrease $\alpha$ by $\alpha _{\text{inc}}$ until $\alpha = \alpha _{\text{min}}$. The parameters are as follows: ${\varepsilon }= 0.01$ for all the panels, $x_1 = 1.0,\, x_2 = 1.5,\, \alpha _{\text{min}} = 0.01,\, \alpha _{\text{max}} = 0.2,$ and $\alpha _{\text{inc}} = 0.01$ for panel (**c**), $\alpha _{\text{min}} = 0.01,\, \alpha _{\text{max}} = 0.2,$ and $\alpha _{\text{inc}} = 0.01$ for panel (**f**), and $a=4,\, b=1,\, \alpha _{\text{min}} = 0.1,\, \alpha _{\text{max}} = 2.0,$ and $\alpha _{\text{inc}} = 0.1$ for panel (**i**). The initial conditions for the first simulation in these three panels are the same ($(x(0), \dot{x}(0)) = (2.0, 0)$). For the following simulations, we use the fixed point of the previous simulation as the initial condition.

We use the following piecewise linear function, which has been previously used to model the escapement mechanism of mechanical clocks^19,26,30^:

13 $$\begin{aligned} g(x, y) {:}{=}{\left\{ \begin{array}{ll} 1 &{} \text{if}\quad x_1< x< x_2, \ y> 0, \\ -1 &{} \text{if}\quad -x_1> x > -x_2, \ y < 0, \\ 0 &{} \text{otherwise}, \end{array}\right. } \end{aligned}$$

where $x_1$ and $x_2$ are positive constants with $x_1 < x_2$. The shape of the function (13) is shown in Fig. 2a.

In this case, the averaging Eq. (11) is calculated as follows:

14 $$\begin{aligned} {\dot{r}}=\left\{ \begin{array}{llll} &{} -\frac{{\varepsilon }\alpha }{2} r &{}\quad \text{if} \quad r< x_1, &{} \qquad \qquad \qquad \qquad \qquad \qquad \\ &{} -\frac{{\varepsilon }\alpha }{2} r + \frac{{\varepsilon }}{\pi } \left( 1-\frac{x_1}{r} \right) &{}\quad \text{if}\quad x_1 \le r < x_2, &{}\qquad \qquad \qquad \qquad \qquad \qquad \\ &{} -\frac{{\varepsilon }\alpha }{2} r + \frac{{\varepsilon }}{\pi } \left( \frac{x_2 - x_1}{r} \right) &{}\quad \text{if} \quad r \ge x_2, &{}\qquad \qquad \qquad \qquad \qquad \qquad \\ \end{array} \right. \end{aligned}$$

15 $$\begin{aligned} r {{\dot{\theta }}} =\left\{ \begin{array}{lll} &{} 0 \quad \text{if} \, r< x_1, &{}\qquad \qquad \qquad \qquad \qquad \qquad \\ &{} -\textstyle \frac{{\varepsilon }}{\pi } \sqrt{1-\frac{x_1^2}{r^2}} \quad \text{if} \, x_1 \le r < x_2, &{}\qquad \qquad \qquad \qquad \qquad \qquad \\ &{} -\textstyle \frac{{\varepsilon }}{\pi } \left( \sqrt{1-\textstyle \frac{x_1^2}{r^2}} - \sqrt{1-\textstyle \frac{x_2^2}{r^2}} \right) \quad \text{if} \, r \ge x_2.&{}\qquad \qquad \qquad \qquad \qquad \qquad \\ \end{array} \right. \end{aligned}$$

See Supplementary Information for the derivation of Eqs. (14) and (15).

Note that Eq. (14) is closed with respect to *r*. By performing a stability analysis of Eq. (14), we find the following:

- Equation (14) has a trivial fixed point $r=0$, which is always stable for any value of $\alpha$.
- The saddle-node bifurcation occurs at
  16 $$\begin{aligned} \alpha = \alpha _{\text{SN}} {:}{=}{\left\{ \begin{array}{ll} \frac{2(x_2 - x_1)}{\pi x_2^2} &{} \text{if} \quad x_2 \le 2x_1, \\ \frac{1}{2\pi x_1} &{} \text{if} \quad x_2 > 2x_1. \end{array}\right. } \end{aligned}$$
- If $\alpha < \alpha _{\text{SN}}$, Eq. (14) has two non-zero fixed points, one stable and the other unstable. The values of these fixed points are given by
  17 $$\begin{aligned} r_{\text{stable}} =\left\{ \begin{array}{lll} &{}\textstyle \frac{1 + \sqrt{1-2\alpha \pi x_1}}{\alpha \pi } \quad \text{if} \quad x_2 > 2x_1, \quad \alpha \ge \textstyle \frac{2(x_2 - x_1)}{\pi x_2^2}, &{}\qquad \qquad \qquad \qquad \qquad \qquad \\ &{}\textstyle \sqrt{\frac{2(x_2 - x_1)}{\alpha \pi }} \quad \text{otherwise}, &{}\qquad \qquad \qquad \qquad \qquad \qquad \\ \end{array} \right. \end{aligned}$$
  18 $$\begin{aligned} r_{\text{unstable}} = \frac{1 - \sqrt{1-2\alpha \pi x_1}}{\alpha \pi }. \end{aligned}$$

See Supplementary Information for the details of the stability analysis. Figure 2b presents the typical flows described by Eq. (14), which shows that the saddle-node bifurcation occurs as $\alpha$ changes. The bifurcation diagram for *r* is shown in Fig. 2c. The green cross marks in Fig. 2c show the numerically obtained equilibrium states of Eq. (6) when we increase $\alpha$, whereas the red dots are those when we decrease $\alpha$. These numerical results agree with the analytically obtained bifurcation diagram, i.e. the black lines in Fig. 2c where we plot Eqs. (17) and (18). Based on this agreement, we consider that our analysis with an averaging approximation is validated.

Figure 2c indicates that our system is bistable for a sufficiently small $\alpha$. Namely, both the oscillatory state ($r=r_{\text{stable}}$) and the resting state ($r=0$) are stable. In addition, by increasing $\alpha$, we see that the stable limit cycle disappears by the saddle-node bifurcation, leading to a state transition from oscillatory to resting state. Therefore, Model (i) reproduces both the bistability and oscillation quenching observed in a real metronome on a movable platform. However, in exchange for the simplicity of the model, the flow becomes non-smooth, which will actually hamper the analysis of synchronization for two metronomes. Motivated by this fact, we also consider smooth functions as *g*(*x*, *y*) below.

#### Model (ii)

We consider

19 $$\begin{aligned} g(x, y) {:}{=}{\left\{ \begin{array}{ll} \frac{x^3}{1+x^4} &{} \text{if}\quad x y > 0, \\ 0 &{} \text{otherwise}. \end{array}\right. } \end{aligned}$$

The shape of the function (19) is shown in Fig. 2d. In this case, the averaging Eq. (11) are calculated as below:

20a $$\begin{aligned} \dot{r}&= -\frac{{\varepsilon }\alpha }{2} r + \frac{{\varepsilon }}{4\pi r} \log (1+r^4), \end{aligned}$$

20b $$\begin{aligned} r {{\dot{\theta }}}&= -\frac{{\varepsilon }}{2r}\left( 1 - \sqrt{ \frac{1+\sqrt{1+r^4}}{2(1+r^4)}}\right) . \end{aligned}$$

See Supplementary Information for the derivation of Eqs. (20a) and (20b).

We consider the dynamics of Eq. (20a). The fixed point of Eq. (20a) satisfies the following transcendental equation:

21 $$\begin{aligned} 2 \pi \alpha R = \log (1+R^2), \end{aligned}$$

where $R {:}{=}r^2$. As shown in Fig. S3 in Supplementary Information, the right-hand side of Eq. (21) is a sigmoid function of *R*. Thus, we see that Eq. (21) has a unique solution (i.e. $R=0$) if $\alpha > \alpha _{\text{SN}}$ and three solutions if $\alpha < \alpha _{\text{SN}}$, where $\alpha _{\text{SN}}$ is given as

22 $$\begin{aligned} \alpha _{\text{SN}} {:}{=}\frac{R^*}{\pi (1+R^{*2})}, \end{aligned}$$

where $R^*$ is a positive constant that satisfies

23 $$\begin{aligned} \frac{2 R^{*2}}{1 + R^{*2}} = \log (1+R^{*2}). \end{aligned}$$

Because there exists a one-to-one relationship between *r* and *R*, we find the following:

- Equation (20a) has a trivial fixed point $r=0$, which is always stable for any value of $\alpha$.
- The saddle-node bifurcation occurs at $\alpha = \alpha _{\text{SN}}$.
- If $\alpha < \alpha _{\text{SN}}$, Eq. (20a) has non-trivial two fixed points, one stable ($r=r_{\text{stable}}$) and the other unstable($r=r_{\text{unstable}}$). The values of these fixed points are the solutions of Eq. (21) with $r_{\text{stable}} > r_{\text{unstable}}$.

Figure 2e shows the typical flows of Eq. (20a) before and after the bifurcation point. The bifurcation diagram for *r* is shown in Fig. 2f, where we see that the numerically obtained equilibrium states of Eq. (6) (green cross marks and red dots) are in good agreement with the analytically obtained bifurcation diagram (black lines).

Figure 2f indicates that Model (ii) also reproduces the bistability and oscillation quenching of a metronome. However, although the flow becomes smooth in this model, we expect that the analysis of the two-oscillator system will be difficult because the averaged system (20) includes the log function and the amplitude of the stable limit cycle, or $r_{\text{stable}}$, cannot be analytically obtained. To further facilitate the analysis, we next use the polynomial function of order 5, which partly imitates Models (i) and (ii) (see Fig. 2a,d and g)).

#### Model (iii)

We consider

24 $$\begin{aligned} g(x, y) {:}{=}{\left\{ \begin{array}{ll} a x^3 - b x^5 &{} \text{if}\quad x y > 0, \\ 0 &{} \text{otherwise}, \end{array}\right. } \end{aligned}$$

where *a* and *b* are positive constants. The shape of the function (24) is shown in Fig. 2g. In this case, the averaging Eq. (11) is calculated as

25a $$\begin{aligned} \dot{r}&= \frac{{\varepsilon }}{12 \pi } \left( - 6 \pi \alpha r +3a r^3 - 2b r^5 \right) , \end{aligned}$$

25b $$\begin{aligned} r {{\dot{\theta }}}&= -{\varepsilon }\left( \frac{3a}{16}r^3 - \frac{5b}{32}r^5 \right) . \end{aligned}$$

See Supplementary Information for the derivation of Eqs. (25a) and (25b).

We discuss the dynamics of Eq. (25a). The fixed point of Eq. (25a) satisfies $6 \pi \alpha r - 3a r^3 + 2b r^5 = 0$, which is solved as

26 $$\begin{aligned} r = 0, \sqrt{\frac{3a \pm \sqrt{9 a^2 - 48 \pi b \alpha }}{4b}}. \end{aligned}$$

By considering the flows of Eq. (25a) as shown in Fig. 2h, we find the following:

- Equation (25a) has a trivial fixed point $r=0$, which is always stable for any value of $\alpha$.
- The saddle-node bifurcation occurs at
  27 $$\begin{aligned} \alpha = \alpha _{\text{SN}} {:}{=}\frac{3 a^2}{16 \pi b}. \end{aligned}$$
- If $\alpha < \alpha _{\text{SN}}$, Eq. (25a) has non-trivial two fixed points, one stable and the other unstable. The values of these fixed points are, respectively, given by
  28 $$\begin{aligned} r_{\text{stable}}&= \sqrt{\frac{3a + \sqrt{9 a^2 - 48 \pi b \alpha }}{4b}}, \end{aligned}$$
  29 $$\begin{aligned} r_{\text{unstable}}&= \sqrt{\frac{3a - \sqrt{9 a^2 - 48 \pi b \alpha }}{4b}}. \end{aligned}$$

The bifurcation diagram for *r* is shown in Fig. 2i. As with Models (i) and (ii), the numerically obtained equilibrium states of Eq. (6) (green cross marks and red dots) are in good agreement with the analytically obtained bifurcation diagram (black lines), which validates our analysis with an averaging approximation.

Figure 2i indicates that Model (iii) reproduces the bistability and oscillation quenching of a metronome. Moreover, the averaged Eqs. (25a) and (25b) are expressed by the polynomial of *r*, which we expect will facilitate the analysis of the two-oscillator system.

## Two metronomes

### Model

**Figure 3:**
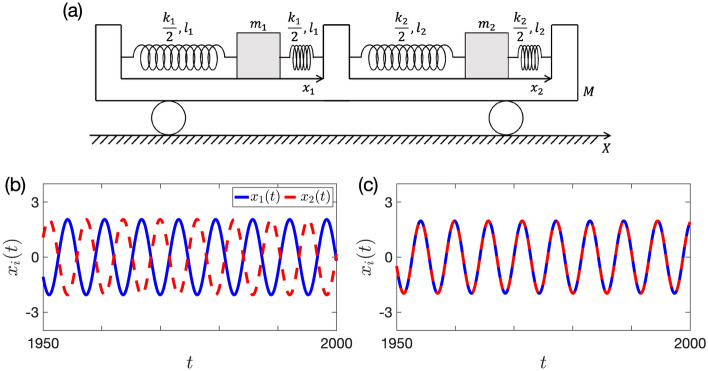
(**a**) The model of two coupled metronomes on a movable platform. (**b**,**c**) Typical dynamics of the nondimensional motion Eq. (37). Anti-phase and in-phase synchronization are observed in panels (**b**) and (**c**), respectively. We set *g* as Eq. (24), ${\varepsilon }= 0.01$, $a=4$, $b=1$, $\mu = 10$, and $\beta = 0.8$. Initial conditions are $(x_1(0),\dot{x}_1(0), x_2(0),\dot{x}_2(0)) = (2.8,0,-2.7,0)$ for panel (**b**) and $(x_1(0),\dot{x}_1(0), x_2(0),\dot{x}_2(0)) = (2.8,0,2.7,0)$ for panel (**c**).

We analyze the synchronization and oscillation quenching of two metronomes on a movable platform, using Model (iii) as the escapement mechanism. The model for the two-oscillator system is shown in Fig. 3a. Here, a point mass $m_i$ ($i=1$ or 2) is connected by two springs with spring constant $k_i/2$ and natural length $l_i$ to a platform of mass *M*. The platform has one degree of freedom and moves freely. The variables *X*(*t*) and $x_i(t)$ are the positions of the platform (more precisely, the position of the platform’s center plate that separates the two point masses) relative to the floor and the mass relative to the platform, respectively. The origin of $x_i$ coordinate is set to the position of the point mass $m_i$ in the equilibrium (i.e. $\dot{x}_1 = \dot{x}_2 = \dot{X}=0$).

By assuming the same situation as in the single metronome model, we obtain the following motion equations:

30 $$\begin{aligned} m_i \ddot{x}_i + k_i x_i + \gamma _i \dot{x}_i - \delta _i f_i(x_i,\dot{x}_i) + m_i {\ddot{X}} = 0, \end{aligned}$$

for $i=1,2$, where $\gamma _i$ is the damping coefficient, $\delta _i$ represents the magnitude of the escapement mechanism, and $f_i(x,{\dot{x}})$ is a dimensionless function that shows the nature of the escapement.

Let $x_\text{c}$ be the position of the center of mass of the whole system. Then,

31 $$\begin{aligned} x_{\text{c}} = \frac{MX + m_1(X-l_1+x_1) + m_2(X + l_2 +x_2)}{M+m_1+m_2}. \end{aligned}$$

Since we assume that there is no external force acting on the whole system, the equation $\frac{d^2}{d t^2}x_\text{c} = 0$ holds, which implies that

32 $$\begin{aligned} {\ddot{X}} = -\frac{m_1 \ddot{x}_1 + m_2 \ddot{x}_2}{M+m_1+m_2}. \end{aligned}$$

Substituting Eq. (32) into Eq. (30), we have

33a $$\begin{aligned} \frac{m_1(M+m_2)}{M+m_1+m_2} \ddot{x}_1 + k_1 x_1 + \gamma _1 \dot{x}_1 - \delta _1 f_1(x_1,\dot{x}_1) -\frac{m_1 m_2}{M+m_1+m_2} \ddot{x}_2 = 0, \end{aligned}$$

33b $$\begin{aligned} \frac{m_2 (M+m_1)}{M+m_1+m_2} \ddot{x}_2 + k_2 x_2 + \gamma _2 \dot{x}_2 - \delta _2 f_2(x_2,\dot{x}_2) -\frac{m_1 m_2}{M+m_1+m_2} \ddot{x}_1 = 0. \end{aligned}$$

We introduce a small dimensionless parameter ${\varepsilon }$ and the following quantities:

34 $$\begin{aligned} \omega {:}{=}\sqrt{\frac{k_1}{m_1}},\, \tau {:}{=}\omega t,\, \beta _i {:}{=}\frac{\omega \gamma _i}{{\varepsilon }k_i},\, \mu _i {:}{=}\frac{m_i}{{\varepsilon }M},\, {{\hat{x}}}_i {:}{=}\frac{k_i x_i}{\delta _i},\, \kappa {:}{=}\frac{k_2}{k_1},\, \rho {:}{=}\frac{\delta _2}{\delta _1}, \, g_i\left( {{\hat{x}}}_i, \frac{d{{\hat{x}}}_i}{d\tau }\right) {:}{=}\frac{1}{{\varepsilon }}f_i\left( \frac{\delta _i {{\hat{x}}}_i}{k_i}, \frac{\omega \delta _i}{k_i}\frac{d{{\hat{x}}}_i}{d\tau }\right) . \end{aligned}$$

By renaming $\tau \rightarrow t$ and ${{\hat{x}}}_i \rightarrow x_i$, we transform Eq. (33) into the following dimensionless system:

35a $$\begin{aligned} \ddot{x}_1 + x_1&= {\varepsilon }\left( -\beta _1 \dot{x}_1 + g_1(x_1,\dot{x}_1) + \frac{\mu _1 \ddot{x}_1 + \frac{\rho }{\kappa } \mu _2 \ddot{x}_2}{1+{\varepsilon }\mu _1 + {\varepsilon }\mu _2}\right) , \end{aligned}$$

35b $$\begin{aligned} \frac{\mu _2}{\mu _1} \ddot{x}_2 + \kappa x_2&= {\varepsilon }\left[ - \kappa \beta _2 \dot{x}_2 + \kappa g_2(x_2,\dot{x}_2) + \frac{\mu _2(\frac{\kappa }{\rho } \mu _1 \ddot{x}_1 + \mu _2 \ddot{x}_2 )}{\mu _1(1+ {\varepsilon }\mu _1 + {\varepsilon }\mu _2)} \right] , \end{aligned}$$

where we assume that $x_i = O(1), \dot{x}_i = O(1), \mu _i = O(1), \beta _i = O(1), \kappa = O(1), \rho = O(1)$, and $g_i(x_i,\dot{x}_i) = O(1)$.

For simplicity, we consider the case where two metronomes are identical. Namely, we set $\mu _1=\mu _2=\mu ,\, \beta _1=\beta _2=\beta ,\, \kappa = \rho = 1$, and $g_1(x,\dot{x})=g_2(x,\dot{x})=g(x,\dot{x})$. Then, Eq. (35) becomes

36 $$\begin{aligned} \ddot{x}_i + x_i = {\varepsilon }\left[ -\beta \dot{x}_i + g(x_i,\dot{x}_i) + \frac{\mu (\ddot{x}_1 + \ddot{x}_2)}{1+ 2 {\varepsilon }\mu } \right] . \end{aligned}$$

Removing $\ddot{x}_1$ and $\ddot{x}_2$ terms from right-hand side of Eq. (36), we can rewrite Eq. (36) as

37 $$\begin{aligned} \ddot{x}_i = - x_i - {\varepsilon }\left[ \mu (x_1 + x_2) + \beta \dot{x}_i - g(x_i,\dot{x}_i) \right] + {\varepsilon }^2 \mu \left[ -\beta (\dot{x}_1 + \dot{x}_2) + g(x_1,\dot{x}_1) + g(x_2,\dot{x}_2) \right] . \end{aligned}$$

The typical behaviors of Eq. (37) are shown in Fig. 3b and c. By neglecting the $O({\varepsilon }^2)$ term from the right-hand side of Eq. (37), we finally obtain

38 $$\begin{aligned} \ddot{x}_i + x_i = - {\varepsilon }\left[ \mu (x_1 + x_2) + \beta \dot{x}_i - g(x_i,\dot{x}_i) \right] . \end{aligned}$$

### Analysis

#### Averaging approximation

We analyze Eq. (38) with an averaging approximation. We rewrite Eq. (38) as

39a $$\begin{aligned} \dot{x}_i&= y_i, \end{aligned}$$

39b $$\begin{aligned} \dot{y}_i&= -x_i - {\varepsilon }\left[ \mu (x_1 + x_2) + \beta y_i - g(x_i,y_i) \right] . \end{aligned}$$

We transform the variables $x_i(t)$ and $y_i(t)$ into the new variables $r_i(t)$, $\theta _i(t)$, and $\phi _i(t)$ that satisfy $r_i(t) \ge 0$ and the following equations:

40a $$\begin{aligned} x_i(t)&= r_i(t) \cos \phi _i(t), \end{aligned}$$

40b $$\begin{aligned} y_i(t)&= - r_i(t) \sin \phi _i(t), \end{aligned}$$

where

41 $$\begin{aligned} \phi _i(t) {:}{=}t + \theta _i(t). \end{aligned}$$

Then, Eq. (39) is transformed into

42a $$\begin{aligned} \dot{r}_i&= {\varepsilon }\sin \phi _i \left[ \mu (r_1 \cos \phi _1 + r_2 \cos \phi _2) - \beta r_i \sin \phi _i - g(r_i \cos \phi _i, -r_i \sin \phi _i) \right] , \end{aligned}$$

42b $$\begin{aligned} {{\dot{\theta }}}_i&= \frac{{\varepsilon }}{r_i} \cos \phi _i \left[ \mu (r_1 \cos \phi _1 + r_2 \cos \phi _2) - \beta r_i \sin \phi _i - g(r_i \cos \phi _i, -r_i \sin \phi _i) \right] . \end{aligned}$$

See Supplementary Information for the derivation of Eq. (42).

Since the estimate $r_i(t) = O(1)$ follows from the assumptions $x_i(t) = O(1)$ and $y_i(t) = O(1)$, Eq. (42) suggests that the time-scale of $r_i(t)$ and $\theta _i(t)$ are $O({\varepsilon }^{-1})$ and thus much larger than the time-scale of metronome’s oscillation period $2\pi$ (i.e. the time-scale of $x_i(t)$ in Eq. (40a)). Therefore, we can safely replace $\dot{r}_i$ and ${{\dot{\theta }}}_i$ with their time average over $2 \pi$ as below:

43a $$\begin{aligned} \dot{r}_1&\simeq \frac{1}{2\pi }\int _0^{2\pi } \dot{r}_1 dt = {\varepsilon }\biggl (\frac{\mu r_2}{2} \sin (\theta _1 - \theta _2) - \frac{\beta r_1}{2} - {{\bar{g}}}_1(r_1) \biggr ), \end{aligned}$$

43b $$\begin{aligned} \dot{r}_2&\simeq \frac{1}{2\pi }\int _0^{2\pi } \dot{r}_2 dt = {\varepsilon }\biggl (\frac{\mu r_1}{2} \sin (\theta _2 - \theta _1) - \frac{\beta r_2}{2} - {{\bar{g}}}_1(r_2) \biggr ), \end{aligned}$$

43c $$\begin{aligned} {{\dot{\theta }}}_1&\simeq \frac{1}{2\pi }\int _0^{2\pi } {{\dot{\theta }}}_1 dt = {\varepsilon }\biggl (\frac{\mu }{2} + \frac{\mu r_2}{2 r_1} \cos (\theta _1 - \theta _2) - \frac{1}{r_1} {{\bar{g}}}_2(r_1) \biggr ), \end{aligned}$$

43d $$\begin{aligned} {{\dot{\theta }}}_2&\simeq \frac{1}{2\pi }\int _0^{2\pi } {{\dot{\theta }}}_2 dt = {\varepsilon }\biggl (\frac{\mu }{2} + \frac{\mu r_1}{2 r_2} \cos (\theta _2 - \theta _1) - \frac{1}{r_2} {{\bar{g}}}_2(r_2) \biggr ), \end{aligned}$$

where the functions $\bar g_{i}(\cdot )\; (i=1,2)$ are given by Eq. (12). Here, based on the same arguments as in the one-oscillator system, we regard $r_i$ and $\theta _i$ as constants when we calculate the integrals in Eq. (43). Several integral formulae used for the derivation of Eq. (43) are summarized in Supplementary Information. Hereafter, we consider the approximately equal sign ($\simeq$) in Eq. (43) as the equal sign ($=$).

By introducing

44 $$\begin{aligned} \psi {:}{=}\theta _2 - \theta _1, \end{aligned}$$

Eq. (43) is rewritten as

45a $$\begin{aligned} \dot{r}_1&= {\varepsilon }\biggl (- \frac{\mu r_2}{2} \sin \psi - \frac{\beta r_1}{2} - {{\bar{g}}}_1(r_1) \biggr ), \end{aligned}$$

45b $$\begin{aligned} \dot{r}_2&= {\varepsilon }\biggl (\frac{\mu r_1}{2} \sin \psi - \frac{\beta r_2}{2} - {{\bar{g}}}_1(r_2) \biggr ), \end{aligned}$$

45c $$\begin{aligned} {{\dot{\psi }}}&= {\varepsilon }\biggl [\frac{\mu }{2} \left( \frac{r_1}{r_2} - \frac{r_2}{r_1} \right) \cos \psi + \frac{1}{r_1} {{\bar{g}}}_2(r_1) - \frac{1}{r_2} {{\bar{g}}}_2(r_2) \biggr ]. \end{aligned}$$

In the analysis of the two-oscillator system, we express the escapement mechanism with Model (iii) because this model exhibits both the reproducibility of actual metronome behavior and the ease of analysis. Then, we finally obtain

46a $$\begin{aligned} \dot{r}_1&= \frac{{\varepsilon }}{12 \pi } \biggl (- 6 \pi \mu r_2 \sin \psi - 6 \pi \beta r_1 + 3a r_1^3 - 2b r_1^5 \biggr ), \end{aligned}$$

46b $$\begin{aligned} \dot{r}_2&= \frac{{\varepsilon }}{12 \pi } \biggl (6 \pi \mu r_1 \sin \psi - 6 \pi \beta r_2 + 3a r_2^3 - 2b r_2^5 \biggr ), \end{aligned}$$

46c $$\begin{aligned} {{\dot{\psi }}}&= \frac{{\varepsilon }}{32} \biggl [16 \mu \left( \frac{r_1}{r_2} - \frac{r_2}{r_1} \right) \cos \psi + 6a(r_1^2 - r_2^2) - 5b(r_1^4 - r_2^4)\biggr ]. \end{aligned}$$

#### Analysis of synchronized states

We perform a linear stability analysis for the averaged system (46). Equation (46) has the following two fixed points

47 $$\begin{aligned} (r_1, r_2, \psi ) = (r^*, r^*, 0), \end{aligned}$$

and

48 $$\begin{aligned} (r_1, r_2, \psi ) = (r^*, r^*, \pi ), \end{aligned}$$

where

49 $$\begin{aligned} r^* {:}{=}\sqrt{\frac{3a + \sqrt{9 a^2 - 48 \pi b \beta }}{4b}}. \end{aligned}$$

Equations (47) and (48) correspond to the in-phase and anti-phase synchronization states, respectively. Note that the condition

50 $$\begin{aligned} \beta < \beta _{\text{SN}} {:}{=}\frac{3a^2}{16\pi b} \end{aligned}$$

is necessary for the existence of these fixed points. Namely, if $\beta \ge \beta _{\text{SN}}$, these fixed points disappear with their counterparts (i.e. the fixed points $\left( \sqrt{\frac{3a - \sqrt{9 a^2 - 48 \pi b \beta }}{4b}}, \sqrt{\frac{3a - \sqrt{9 a^2 - 48 \pi b \beta }}{4b}}, 0 \right)$ and $\left( \sqrt{\frac{3a - \sqrt{9 a^2 - 48 \pi b \beta }}{4b}}, \sqrt{\frac{3a - \sqrt{9 a^2 - 48 \pi b \beta }}{4b}}, \pi \right)$ that pair with Eq. (47) and Eq. (48), respectively) by the saddle-node bifurcation.

By performing a linear stability analysis under the condition (50), we find the following:

1. The fixed point (48), which corresponds to the anti-phase synchrony, is always asymptotically stable.
2. The fixed point (47), which corresponds to the in-phase synchrony, is asymptotically stable if and only if
  51 $$\begin{aligned} \mu > \mu _{\text{c}} {:}{=}- \frac{15 \pi \beta }{8} + \frac{27a^2}{64b} + \frac{9a}{64b}\sqrt{9a^2 - 48 \pi b \beta }. \end{aligned}$$

The details of the stability analysis are summarized in Supplementary Information.

#### Analysis of oscillation quenching

Next, we examine the stability of oscillation quenching (i.e. $x_i=\dot{x}_i=0$) in Eq. (37). In a sufficient neighborhood of oscillation quenching, Eq. (37) can be linearized as

52 $$\begin{aligned} \ddot{x}_i = - x_i - {\varepsilon }\left[ \mu (x_1 + x_2) + \beta \dot{x}_i \right] - {\varepsilon }^2 \mu \beta (\dot{x}_1 + \dot{x}_2). \end{aligned}$$

By introducing $z_{\text{c}} {:}{=}x_1+x_2$ and $z_{\text{r}} {:}{=}x_1-x_2$, Eq. (52) becomes

53a $$\begin{aligned}&\ddot{z}_{\text{c}} + (1 + 2{\varepsilon }\mu ) z_{\text{c}} = - {\varepsilon }\beta (1+{\varepsilon }^2 \mu ) \dot{z}_{\text{c}}, \end{aligned}$$

53b $$\begin{aligned}&\ddot{z}_{\text{r}} + z_{\text{r}} = - {\varepsilon }\beta \dot{z}_{\text{r}}. \end{aligned}$$

Since Eqs. (53a) and (53b) are both the equations of damped oscillation, we see that the fixed point $z_{\text{c}} = \dot{z}_{\text{c}} = z_{\text{r}} = \dot{z}_{\text{r}} =0$, which corresponds to oscillation quenching, is asymptotically stable. In other words, oscillation quenching is always stable for any values of $\mu$ and $\beta$. Therefore, we expect that the transition from synchronization to oscillation quenching occurs if

54 $$\begin{aligned} \beta > \beta _{\text{SN}}, \end{aligned}$$

because in this region the fixed points that correspond to the synchronous states (i.e. Eqs. (47) and (48)) do not exist.

As is shown below, the parameter space in which oscillation quenching occurs is wider than the inequality (54) if the two metronomes are in-phase synchronized. By assuming that $x_1 = x_2 = x$, the original motion equation (36) is transformed into

55 $$\begin{aligned} \frac{1}{1+2{\varepsilon }\mu }\ddot{x} + x = {\varepsilon }(-\beta \dot{x} + g(x,\dot{x})). \end{aligned}$$

By setting $\tau {:}{=}t \sqrt{1+2{\varepsilon }\mu }$ and renaming $\tau \rightarrow t$, we obtain

56 $$\begin{aligned} \ddot{x} + x = {\varepsilon }(-\beta \sqrt{1+2{\varepsilon }\mu } \dot{x} + g(x,\dot{x})). \end{aligned}$$

Note that $g(x,\dot{x}) = g(x,\dot{x} \sqrt{1+2{\varepsilon }\mu })$ when the function *g* is given by Eq. (24). In the analysis of Eq. (56), we can remove the assumption that $\mu = O(1)$ because Eq. (56) is considered as a weakly nonlinear oscillator as long as ${\varepsilon }\mu = O(1)$.

An averaging approximation of Eq. (56) yields

57a $$\begin{aligned} \dot{r}&= \frac{{\varepsilon }}{12 \pi } \left( - 6 \pi \beta \sqrt{1+2{\varepsilon }\mu } r +3a r^3 - 2b r^5 \right) , \end{aligned}$$

57b $$\begin{aligned} r {{\dot{\theta }}}&= -{\varepsilon }\left( \frac{3a}{16}r^3 - \frac{5b}{32}r^5 \right) , \end{aligned}$$

where *r* and $\theta$ are given by Eqs. (8) and (9). By considering the flows of Eq. (57a), we find that the saddle-node bifurcation occurs when

58 $$\begin{aligned} \beta = \beta _{\mathrm{SN\_in}} {:}{=}\frac{3 a^2}{16\pi b \sqrt{1+2{\varepsilon }\mu }}, \end{aligned}$$

which implies that the transition from in-phase synchronization to oscillation quenching occurs when

59 $$\begin{aligned} \beta > \beta _{\mathrm{SN\_in}}. \end{aligned}$$

Note that the condition (59) is looser than the condition (54). Namely, there exist parameter regions in which oscillation quenching occurs when two metronomes are in-phase synchronized whereas the anti-phase synchronous state is stable. This analytical result agrees with previous experimental observations using pendulum clocks^18^.

**Figure 4:**
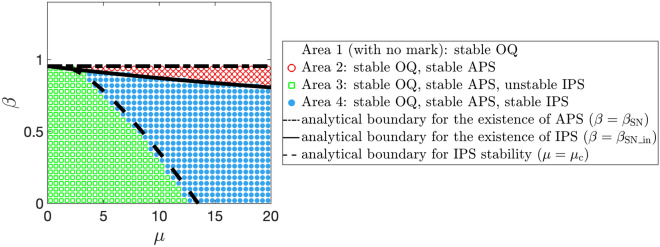
The phase diagram of the system (36) for anti-phase synchronization (APS), in-phase synchronization (IPS), and oscillation quenching (OQ). We use Model (iii) to describe the escapement mechanism. The black lines represent the analytical boundaries obtained by an averaging approximation. The in-phase and anti-phase synchronization disappear by the saddle-node bifurcation at the solid line (given by $\beta = \beta _{\mathrm{SN\_in}}$) and the dash-dotted line (given by $\beta = \beta _{\text{SN}}$), respectively. The stability of in-phase synchronization switches at the dashed line, which is given by $\mu = \mu _{\text{c}}$. The colored marks and white area show the numerical simulation results of Eq. (37). In area 1, where no symbol is plotted, oscillation quenching occurs when we use any of the 4 initial conditions described in the article, suggesting that neither anti-phase nor in-phase synchronization exists. In area 2, which is marked with red circles, anti-phase synchronization is stable, whereas in-phase synchronization does not exist. Namely, oscillation quenching occurs if we use the initial condition that is either exactly or close to the in-phase synchronization. In area 3, which is marked by green square marks, anti-phase synchronization is stable, while in-phase synchronization is unstable. Both anti-phase and in-phase synchronizations are stable in area 4 marked by blue dots. Note that oscillation quenching is stable in all of the 4 areas. We fix $a=4, b=1, {\varepsilon }= 0.01$ in the simulation.

Based on the above existence and stability analyses of synchronization and quenching, we depict the phase diagram, which is shown as the black lines in Fig. 4. The dash-dotted and solid lines are given by $\beta = \beta _{\text{SN}}$ and $\beta = \beta _{\mathrm{SN\_in}}$, respectively. Namely, the former is the boundary of whether oscillation quenching occurs when the two metronomes are anti-phase synchronized, whereas the latter corresponds to that when the two metronomes are in-phase synchronized. The dashed line is given by $\mu = \mu _{\text{c}}$ in inequality (51). In other words, the stability of in-phase synchronization switches at the dashed line.

The solid and dash-dotted lines in Fig. 4 correspond to the saddle-node bifurcation. Since the Jacobian matrix at the fixed point (47) has a zero eigenvalue on the dashed line (see Supplementary Information), this line corresponds to either of the saddle-node, transcritical, or pitchfork bifurcation^37^. According to the symmetry of Eq. (46) (i.e. Eq. (46) is invariant if we change $r_1 \rightarrow r_2,\, r_2 \rightarrow r_1$ and $\psi \rightarrow -\psi$) and the fact that this fixed point (47) does not disappear after the bifurcation, the saddle-node and transcritical bifurcations are unlikely. As we describe later, simulation results suggest that a stable fixed point that satisfies $r_1 \ne r_2$ emerges near the bifurcation point (see Figs. 6 and S5a–c in Supplementary Information). Thus, we consider that the dashed line in Fig. 4 corresponds to the supercritical pitchfork bifurcation.

### Numerical simulation

We verify our analysis with an averaging approximation by numerically integrating Eq. (37). The results of numerical simulations are shown Fig. 4, where we use the following 4 initial conditions:

(i) Near in-phase synchronization ($x_1(0) = 5.01,\, x_2(0) = 5,\, \dot{x}_{1,2} (0) = 0$),
(ii) In-phase synchronization ($x_1(0) = x_2(0) = 5,\, \dot{x}_{1,2} (0) = 0$),
(iii) Near anti-phase synchronization ($x_1(0) = 5.01,\, x_2(0) = -5,\, \dot{x}_{1,2} (0) = 0$),
(iv) Anti-phase synchronization ($x_1(0) = 5,\, x_2(0) = -5,\, \dot{x}_{1,2} (0) = 0$).

In area 1 of Fig. 4, oscillation quenching occurs when we use any of the above 4 initial conditions. In area 2, both the solutions from the initial conditions (iii) and (iv) converge to anti-phase synchronization, which implies that anti-phase synchronization is asymptotically stable. However, oscillation quenching occurs if we use either the initial condition (i) or (ii), suggesting that in-phase synchronization does not exist. In area 3, the solution from the initial condition (ii) converges in-phase synchronization, whereas the solution from the initial condition (i) does not, meaning that in-phase synchronization is an unstable steady state. Anti-phase synchronization is stable in this area. In area 4, both anti-phase and in-phase synchronizations are stable. Note that oscillation quenching is always stable in these 4 areas.

In Fig. 4, we see that the analytically and numerically obtained phase diagrams are in good agreement, which confirms the validity of the averaging approximation used in this study. However, the boundaries for the stability of in-phase synchronization obtained by analysis (the dashed line) and simulation (the boundary between areas 3 and 4) are slightly different. To clarify the cause of this difference, we numerically calculate $\mu _{\text{c}}$ by a bisection method and plot the values for different ${\varepsilon }$ (Fig. S4 in Supplementary Information). Figure S4 shows that numerically obtained $\mu _{\text{c}}$ approaches the analytical value (i.e. $\mu _{\text{c}}$ in inequality (51)) as ${\varepsilon }$ decreases. Thus, we consider that the $O({\varepsilon }^2)$ term which is neglected in the averaging approximation causes the difference between the numerically and analytically obtained boundaries in Fig. 4.

**Figure 5:**
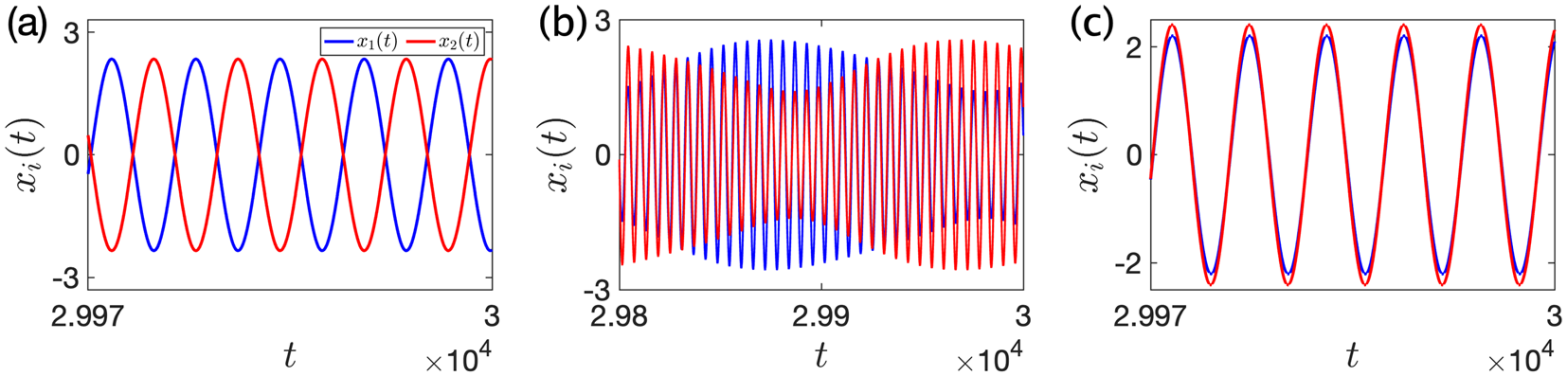
The equilibrium states of Eq. (37) when we use the initial condition (i). We numerically integrate Eq. (37) and plot $x_i(t)$ after a sufficiently long time. We fix $a=4,\, b=1,\, {\varepsilon }= 0.01, \, \beta = 0.3$ in this figure. (**a**) When $\mu = 3$, anti-phase synchronization is observed. (**b**) When $\mu = 6.5$, the amplitude of each metronome alternately increases and decreases. (**c**) When $\mu = 9.5$, near in-phase synchronized movement with slightly different amplitudes is observed. Note that this behavior corresponds to out-of-phase synchronization.

In the parameter region where in-phase synchronization is unstable (i.e. area 3 in Fig. 4), several equilibrium states are numerically observed when we use the initial condition (i). The results are shown in Fig. 5, where we select several sets of parameters $\beta , \mu$ and plot the time series of $x_i(t)$ in Eq. (37) after a sufficiently long time. In our simulation results, either of the anti-phase synchronization (Fig. 5a), the alternating increase and decrease of amplitudes which is similar to the beat phenomena in the acoustic wave (Fig. 5b), and the out-of-phase synchronization with slightly different amplitudes (Fig. 5c) are observed. In particular, the dynamics in Fig. 5b,c suggest that there exist limit cycles and other fixed points than Eqs. (47) and (48) in the averaged system (46).

**Figure 6:**
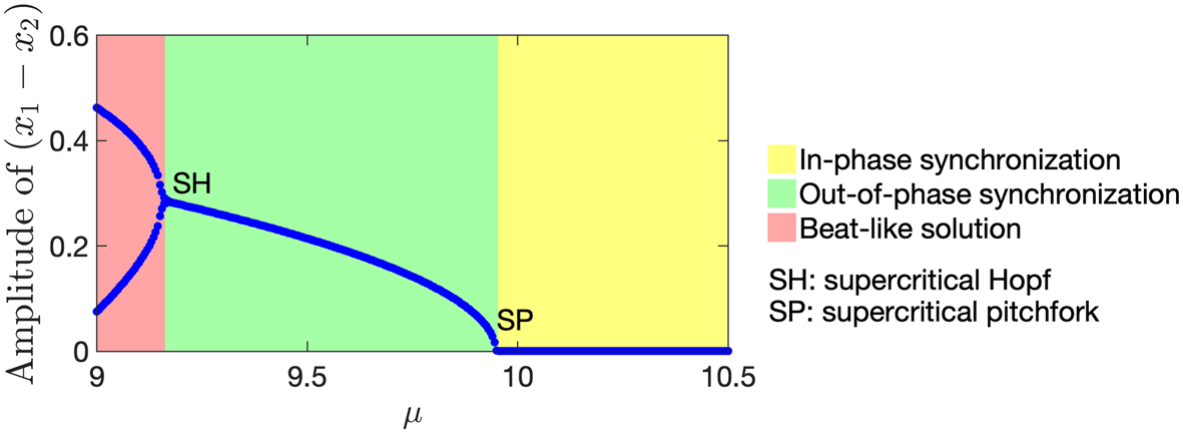
Numerically obtained bifurcation diagram for in-phase synchronization (yellow area), out-of-phase synchronization (green area), and beat-like solution (red area). We plot the amplitude of ($x_1 - x_2$) against different values of $\mu$. Note that we plot both the maximum and minimum values of the amplitude if the beat-like solution is observed (i.e. if the amplitude of ($x_1 - x_2$) varies with time). See Fig. S5 for individual time series that correspond to several values of $\mu$. This figure suggests that out-of-phase synchronization emerges from in-phase synchronization by the supercritical pitchfork bifurcation and the beat-like solution emerges from out-of-phase synchronization by the supercritical Hopf bifurcation. We fix $a = 4,\ b = 1,\ {\varepsilon }= 0.01, \beta = 0.3$. We first simulate the case when $\mu = 10.5$, after which we decrease $\mu$ by 0.005 until $\mu = 9$. We use initial condition (i) for the first simulation. For the following simulations, we use the slightly perturbed equilibrium state of the previous simulation as the initial condition (i.e. $(x_1(0), y_1(0), x_2(0), y_2(0)) = (x_1^{\dagger } + 0.01, y_1^{\dagger }, x_2^{\dagger }, y_2^{\dagger })$ where $(x_1^{\dagger }, y_1^{\dagger }, x_2^{\dagger }, y_2^{\dagger })$ is the equilibrium state of the previous simulation).

Finally, we numerically investigate the bifurcation of the beat-like solution in Fig. 5b. Figure S5d and e show the time course of positional difference of two metronomes ($x_1(t) - x_2(t)$). Figure S5d corresponds to the out-of-phase synchrony state (Fig. 5c), whereas Fig. S5e corresponds to the beat kind of solution (Fig. 5b) because the amplitude of the fast oscillations varies with time. By decreasing $\mu$, we see that the out-of-phase synchrony state (Fig. S5d) becomes unstable and the beat kind of solution (Fig. S5e) emerges. The slow oscillation of the amplitude in Fig. S5e can be more easily detected in Fig. S5f, where we plot the time course of local maximal values of the dynamics in Fig. S5d and e. A more detailed bifurcation diagram is shown in Fig. 6, where we plot the amplitude of ($x_1 - x_2$) after a sufficiently long time against different values of $\mu$. We plot both the maximum and minimum value of the amplitude when it oscillates (like the blue line in Fig. S5f), corresponding to the beat-like solution. According to these arguments and Figs. 6 and S5, we speculate that the beat-like solution in Fig. 5b emerges by the supercritical Hopf bifurcation.

## Discussion

In this study, we investigate the dynamics of a metronome, which is an example of a bistable system with a stable limit cycle and a stable fixed point. In particular, we focus on the oscillation quenching phenomenon observed in metronomes on a movable platform. First, we construct a mathematical model for a single-oscillator system, ignoring the nonlinearity of the pendulum structure and the damping of the platform. By performing the averaging approximation, we consider several functions to represent the escapement mechanism and find that these functions are appropriate for simulating the real metronome’s bistability and oscillation quenching. We conclude that the fifth-order polynomial is the most suitable for both the ease of analysis and reproducibility of the real metronome. Subsequently, we expand our model to include a two-oscillator system. By assuming that the mass ratio of the metronome to the platform, the escapement mechanism, and the damping force are sufficiently small, we perform an averaging approximation and reduce the system to a three-dimensional dynamical system of the amplitudes $r_{1,2}$ and phase difference $\psi$. By performing a stability analysis on the averaged system, we obtain the phase diagram for the in-phase synchronization, anti-phase synchronization, and oscillation quenching. We also numerically integrate the original motion equations and confirm the agreement between the numerically and analytically obtained phase diagrams.

One of the characteristics of this study is that we choose to simplify the model instead of trying to include all the details of the real system. Nevertheless, our model successfully reproduces several experimental behaviors such as the bistability of in-phase and anti-phase synchronization^21^ and the occurrence of oscillation quenching by increasing the mass ratio between the oscillator and the platform^18^. Based on these agreements with experimental results, we believe that our simplification does not impair the essence of real mechanical oscillators.

The analytical method used in this study (i.e. averaging approximation) has been used in previous studies such as those by Pantaleone^17^ and Goldsztein et al.^27,31^ However, oscillation quenching was difficult to analyze in the models adopted in these studies. This is because the former study^17^ used the van der Pol-type function to describe the escapement mechanism, which made the resting state unstable, and the latter studies^27,31^ used the delta function, which restricted the detailed analysis of oscillation quenching. We consider several other functions (i.e. Model (i)-(iii)) for the escapement mechanism and show that the averaged system becomes bistable. In particular, for a fifth-order polynomial (Model (iii)), we can explicitly derive the amplitude of the stable limit cycle (Eq. 28). This is expected to ease the analysis. Thus, we adopt Model (iii) to describe the escapement of the two-oscillator system and successfully obtain the analytical phase diagram including the area where oscillation quenching occurs.

According to a previous study that surveyed in detail the structure of real pendulum clocks^16^, a piecewise linear function is considered appropriate to model the escapement mechanism. In this sense, Model (i) is consistent with reality, although the analysis becomes difficult with this model. To facilitate the analysis, we choose to model the escapement mechanism with smooth functions, especially a 5th-order polynomial (Model (iii)). Although in Model (iii) a large unrealistic negative restoring force acts at a location far from the origin, the bifurcation diagram (Fig. 2i) is qualitatively the same as those of Model (i) (Fig. 2c) when there exists positive damping (i.e. when $\alpha > 0$). Namely, Model (iii) captures two unique features of a real metronome: bistability and oscillation quenching. According to this agreement between model behavior and real dynamics, we consider that the use of a 5th-order polynomial as the escapement mechanism can be justified.

Our study shows that oscillation quenching occurs by a saddle-node bifurcation when the mass ratio $\mu$ increases. This is evident from Eq. (5) in the case of one metronome (note that the bifurcation parameter $\alpha$ increases as $\mu$ increases) and Fig. 4 in the case of two metronomes. As $\mu$ corresponds to the magnitude of the feedback that the metronome receives from the platform, our findings indicate that the oscillation can be stopped by the feedback resulting from the motion of the oscillator.

The occurrence of oscillation quenching by a saddle-node bifurcation was already reported in a previous study^27^ for the case of a metronome on a fixed platform. However, our study reveals that the same bifurcation is observed even for a metronome on a movable platform. Further, we analytically find that oscillation quenching occurs when $\mu$ increases if two metronomes on a movable platform move almost in-phase synchronization, which has not been shown in previous studies^27,31^. In previous experiments with two pendulum clocks suspended from a common plate^18^, it was observed that, when two clocks start from the initial condition close to in-phase synchronization, oscillation quenching occurs as the mass ratio between the pendulum clock and the entire system increases. This observation agrees with our results, which suggests that the simplification in our modeling and the analysis with the averaging approximation are valid.

The contributions of our study, in comparison to the recent relevant studies^27,31^, are summarized as follows. First, by simplifying the modeling of the escapement mechanism, we have performed stability analysis of both synchronization and oscillation quenching of metronomes on a movable platform. In contrast, only the stability of synchronization^31^ and no analytical calculation regarding the stability of steady-state solutions^27^ were addressed in previous studies. Second, we analyze the mechanism behind oscillation quenching of metronomes not on a fixed platform (argued in the previous study^27^) but on a movable platform. Namely, we find that quenching occurs when the stable limit-cycle solution disappears by saddle-node bifurcation. In addition, we clarify that quenching occurs by increasing the mass ratio $\mu$ between the metronome and the platform when two metronomes are almost in-phase synchronization, which agrees with the past experiments using pendulum clocks^18^. We consider that these findings on oscillation quenching are novel because the previous work only investigated the quenching of a metronome on a fixed platform^27^. Third, we find several characteristic model behaviors that have not been analyzed in previous studies^27,31^, i.e. out-of-phase synchronization (Fig. 5c) and beat-like solution (Fig. 5b). We numerically investigate the origin of these two dynamics and obtain a bifurcation diagram (Fig. 6), which we consider is also a novel contribution of our study.

Regarding the state-transition method from a limit cycle to a stable fixed point, our study suggests that an oscillation can be stopped by increasing the feedback resulting from the oscillator. Namely, in our model the magnitude of the feedback depends on the mass ratio $\mu$; thus, oscillations can be controlled by increasing $\mu$ instead of directly intervening in the oscillator. This type of feedback-induced quenching can be applied to the termination of oscillation in other nonlinear bistable systems, such as epilepsy models^2,3,38^ where pathological and normal brain activities are expressed by a limit cycle and a fixed point, respectively. Thus, our study results might lead to a clue for the novel treatment of the disease. In addition, Fig. 4 indicates that any perturbation that switches from anti-phase to in-phase synchronization can evoke oscillation quenching in certain parameter regions (that is, area 2 in Fig. 4). Exploring the same phenomenon, where switching between bistable states causes oscillation quenching, in other coupled oscillator systems is a future challenge because it might help remove undesirable collective oscillations.

There are several open questions in this study. (1) In the analysis of the averaged system (46), we have not performed the existence and stability analysis of equilibrium states other than oscillation quenching and synchronization. Based on our numerical simulation, the beat kind of phenomenon is observed (Fig. 5b), suggesting that the system (46) has a stable limit cycle. We also obtain the out-of-phase synchrony state (Fig. 5c), which indicates that there exists a fixed point that satisfies $r_1 \ne r_2$ in system (46). We consider that the latter state corresponds to the experimentally confirmed phenomenon called “metronome suppression”^27^, in which one metronome oscillates with a larger amplitude than the other. Although we discuss the origin and bifurcation of these solutions by numerically obtained results shown in Figs. 6 and S5, the rigorous bifurcation analysis of the averaged system (46) and, if possible, the original system (36) is an important challenge for future study. (2) When modeling a metronome, we neglect the nonlinearity caused by the pendulum structure. There are two reasons for this simplification: (1) the analysis becomes easier, and (2) such nonlinearity does not change the dynamics of amplitude *r* after the averaging approximation. The second reason is explained as follows. In the model that considers the weak nonlinearity of the pendulum structure^27,31^, term ${\varepsilon }\sigma x^3$ with a real constant $\sigma$ is added to the motion equation as a result of the Taylor expansion of $\sin x$ to the third-order term. Namely, Eq. (7) would be

60a $$\begin{aligned} \dot{x}&= y, \end{aligned}$$

60b $$\begin{aligned} \dot{y}&= -x + {\varepsilon }\left( - \alpha y + \sigma x^3 + g(x,y) \right) , \end{aligned}$$

which implies that

61a $$\begin{aligned} \dot{r}&= - {\varepsilon }\sin \phi \left( \alpha r \sin \phi + \sigma r^3 \cos ^3 \phi + g(r \cos \phi , -r \sin \phi ) \right) , \end{aligned}$$

61b $$\begin{aligned} {{\dot{\theta }}}&= - \frac{{\varepsilon }}{r} \cos \phi \left( \alpha r \sin \phi + \sigma r^3 \cos ^3 \phi + g(r \cos \phi , -r \sin \phi ) \right) . \end{aligned}$$

Then, the averaging approximation in Eq. (61) yields

62a $$\begin{aligned} \dot{r}&= -{\varepsilon }\left( \frac{\alpha r}{2} + {{\bar{g}}}_1(r) \right) , \end{aligned}$$

62b $$\begin{aligned} {{\dot{\theta }}}&= - \frac{{\varepsilon }}{r} \left( \frac{3}{8} \sigma r^3 + {{\bar{g}}}_2(r) \right) . \end{aligned}$$

Comparing Eq. (11) with (62), we see that the dynamics of *r* are the same, whereas the dynamics of $\theta$ change. Because this study mainly addresses the state transition from a limit cycle to a stable fixed point and thus focuses on *r* dynamics, we neglect the nonlinearity of the metronome, which does not change the dynamics of *r*. However, as $\theta$ dynamics are related to the phase of the oscillator, the results of the stability analysis for the two-oscillator system would change if we adopt Eq. (60) as the metronome’s motion equation. Indeed, several previous experiments on coupled metronomes observed that under particular settings in-phase synchronization becomes stable whereas anti-phase synchronization is unstable^17,21^, which cannot be explained in our model. Including the nonlinearity of the pendulum structure in our model might solve this disagreement with past experiments and thus an important research direction for the next study.

## Conclusion

We investigate the dynamics of metronomes on a movable platform using an averaging approximation and numerical simulation. To facilitate the analysis, we ignore the nonlinearity caused by the pendulum structure of the metronome and model the escapement mechanism using a fifth-order polynomial. We obtain a phase diagram and find that oscillation quenching occurs when the mass ratio between the metronome and platform increases, which agrees with previous experimental results using pendulum clocks^18^. The rigorous bifurcation analysis of the averaged system (46) and the refinement of our model by including the nonlinearity of pendulum structure are important challenges for future study. We believe that our simple model will contribute to future analyses of other dynamics of mechanical oscillators, such as clustering^19^, the chimera states^22,23^, and chaotic dynamics^29^.

## Methods

All of the numerical simulations in this article were performed with MATLAB ODE45 solver. Both the absolute and relative tolerances are set to 1e−9 ($10^{-9}$).

### Supplementary Information

Supplementary Information.

## Data Availability

All data generated or analyzed during this study are available from the corresponding author on reasonable request.
