# Supplementary material for: Weakly nonlinear analysis on synchronization and oscillation quenching of coupled mechanical oscillators
Source: Sci Rep. 2024 Jan 17;14:1461. doi: 10.1038/s41598-024-51843-9 (PMC10794449; doi:10.1038/s41598-024-51843-9)
Supplement: Supplementary file 1 — Supplementary Information. [file 41598_2024_51843_MOESM1_ESM.pdf]

Supplementary information for "Weakly nonlinear analysis on synchronization and oscillation quenching of coupled mechanical oscillators"

Yusuke Kato[1,*] and Hiroshi Kori[1]

[1]Department of Complexity Science and Engineering, Graduate School of Frontier Sciences, The University of Tokyo, Kashiwa, Chiba 277-8561, Japan
[*]To whom correspondence should be addressed. Email: yuukato@g.ecc.u-tokyo.ac.jp

December 23, 2023

1 Derivation of Eq. (10)

We introduce the complex variable $A(t)$ that satisfies

$$x(t) = \frac{1}{2}(A(t)e^{it} + \text{c.c.}), \tag{S1a}$$

$$y(t) = \frac{1}{2}(iA(t)e^{it} + \text{c.c.}). \tag{S1b}$$

Note that c.c. denotes the complex conjugate. Then, it follows from Eq. (S1) that

$$A(t) = (x - iy)e^{-it}, \tag{S2}$$

which implies that

$$\dot{A}(t) = [(\dot{x} - y) - i(\dot{y} + x)]\, e^{-it}. \tag{S3}$$

By substituting Eq. (7) into Eq. (S3), we obtain

$$\dot{A} = ie^{-it}\varepsilon\left(\alpha y - g(x, y)\right). \tag{S4}$$

We express $A(t)$ in a polar coordinate as below:

$$A(t) = r(t)e^{i\theta(t)}, \tag{S5}$$

where $r(t), \theta(t)$ are real variables with $r(t) \geq 0$. Then, Eq. (8) follows from Eqs. (S1) and (S5). By substituting Eqs. (S5) and (8) into Eq. (S4), we have

$$\dot{r} + ir\dot{\theta} = -ie^{-i\phi}\varepsilon\left(\alpha r \sin\phi + g(r\cos\phi, -r\sin\phi)\right), \tag{S6}$$

and thus we obtain Eq. (10).

2 Several integral formulae with trigonometric functions

In the derivation of Eqs. (11) and (43), we use

$$\int_0^{2\pi} d\phi \sin^2\phi = \int_0^{2\pi} d\phi \cos^2\phi = \pi, \tag{S7a}$$

$$\int_0^{2\pi} d\phi \sin\phi\cos\phi = 0, \tag{S7b}$$

and

$$\int_0^{2\pi} \sin\phi_i \cos\phi_j dt = \int_0^{2\pi} \frac{\sin(\phi_i+\phi_j)+\sin(\phi_i-\phi_j)}{2} dt = \pi\sin(\theta_i-\theta_j), \tag{S8}$$

$$\int_0^{2\pi} \sin\phi_i \sin\phi_j dt = \int_0^{2\pi} \frac{\cos(\phi_i-\phi_j)-\cos(\phi_i+\phi_j)}{2} dt = \pi\cos(\theta_i-\theta_j), \tag{S9}$$

$$\int_0^{2\pi} \cos\phi_i \cos\phi_j dt = \int_0^{2\pi} \frac{\cos(\phi_i+\phi_j)+\cos(\phi_i-\phi_j)}{2} dt = \pi\cos(\theta_i-\theta_j), \tag{S10}$$

for $i \neq j$. Remind that ϕ_i is given by Eq. (41) and θ_i is regarded as constant during the integral.

3 Calculation of $\bar{g}_{1,2}(r)$ in the case where g is given as Eq. (13)

By substituting Eq. (8) into Eq. (13), we have

$$g(r\cos\phi, -r\sin\phi) \coloneqq \begin{cases} 1 & \text{if} \quad x_1 < r\cos\phi < x_2,\ r\sin\phi < 0, \\ -1 & \text{if} \quad -x_1 > r\cos\phi > -x_2,\ r\sin\phi > 0, \\ 0 & \text{otherwise.} \end{cases} \tag{S11}$$

We calculate the integrals in Eq. (12) by dividing them into the following three cases: (i) $0 \le r < x_1$, (ii) $r > x_2$, and (iii) $x_1 < r < x_2$.

(i) In the case where $0 \le r < x_1$,

$$g(r\cos\phi, -r\sin\phi) = 0$$

holds over the integral interval (i.e., $\phi \in [0, 2\pi]$), which implies that

$$\bar{g}_1(r) = \bar{g}_2(r) = 0. \tag{S12}$$

(ii) In the case where $r > x_2$, Eq. (S11) can be written as

$$g(r\cos\phi, -r\sin\phi) \coloneqq \begin{cases} 1 & \text{if} \quad 2n\pi - \phi_1 < \phi < 2n\pi - \phi_2, \\ -1 & \text{if} \quad (2n+1)\pi - \phi_1 < \phi < (2n+1)\pi - \phi_2, \\ 0 & \text{otherwise,} \end{cases} \tag{S13}$$

where

$$\phi_i \coloneqq \mathrm{Arccos}\frac{x_i}{r} \tag{S14}$$

and n is an arbitrary integer. Then,

$$\begin{aligned} \bar{g}_1(r) &= \frac{1}{2\pi}\left(\int_{2\pi-\phi_1}^{2\pi-\phi_2} \sin\phi - \int_{\pi-\phi_1}^{\pi-\phi_2} \sin\phi\right) \\ &= \frac{x_1 - x_2}{\pi r}, \end{aligned} \tag{S15a}$$

$$\begin{aligned} \bar{g}_2(r) &= \frac{1}{2\pi}\left(\int_{2\pi-\phi_1}^{2\pi-\phi_2} \cos\phi - \int_{\pi-\phi_1}^{\pi-\phi_2} \cos\phi\right) \\ &= \frac{1}{\pi}\left(\sqrt{1-\frac{x_1^2}{r^2}} - \sqrt{1-\frac{x_2^2}{r^2}}\right). \end{aligned} \tag{S15b}$$

(iii) The case where $r > x_2$ corresponds to the special case of the previous case: we only have to substitute 0 into ϕ_2 (i.e., to substitute r into x_2) in Eqs. (S13) and (S15). Thus,

$$\bar{g}_1(r) = 2\left(\frac{x_1}{r} - 1\right), \tag{S16a}$$

$$\bar{g}_2(r) = 2\sqrt{1-\frac{x_1^2}{r^2}}. \tag{S16b}$$

4 Stablity analysis of Eq. (14)

Obviously, Eq. (14) has a trivial fixed point $r = 0$, which is stable because $\dot{r} < 0$ holds in a sufficient neighborhood of $r = 0$. By differentiating the right-hand-side of Eq. (14) by r, we obtain

$$\frac{d\dot{r}}{dr} = \begin{cases} -\frac{\varepsilon\alpha}{2} & \text{if} \quad r < x_1, \\ -\varepsilon\left(\frac{\alpha}{2} - \frac{x_1}{\pi r^2}\right) & \text{if} \quad x_1 \le r < x_2, \\ -\frac{\varepsilon\alpha}{2} - \frac{\varepsilon(x_2 - x_1)}{\pi r^2} & \text{if} \quad r \ge x_2. \end{cases} \tag{S17}$$

It is easily seen that $\frac{d\dot{r}}{dr} < 0$ if $r < x_1$ or $r \ge x_2$, which implies that $\dot{r}$ is monotonically decreasing function of r in these intervals. We also find that $\frac{d\dot{r}}{dr} = 0$ holds if and only if $r = \sqrt{\frac{2x_1}{\pi\alpha}}$ and $x_1 < \sqrt{\frac{2x_1}{\pi\alpha}} < x_2$. Below, we consider the increase or decrease of $\dot{r}$ in the interval $x_1 \le r < x_2$ by dividing the cases whether $\sqrt{\frac{2x_1}{\pi\alpha}}$ belongs to this interval.

(i) We first consider the case where $\sqrt{\frac{2x_1}{\pi\alpha}} < x_1$, which is equivalent to

$$\alpha > \frac{2}{\pi x_1}. \tag{S18}$$

In this case, $\frac{d\dot{r}}{dr} < 0$ when $x_1 \le r < x_2$, which implies that $\dot{r}$ is a monotonically decreasing function of r for the whole interval $r \in [0, \infty)$. Thus, $r = 0$ is the only fixed point of Eq. (14).

(ii) Next, we consider the case where $x_1 \le \sqrt{\frac{2x_1}{\pi\alpha}} < x_2$, which is equivalent to

$$\frac{2x_1}{\pi x_2^2} < \alpha \le \frac{2}{\pi x_1}. \tag{S19}$$

In this case,

$$\begin{aligned} \max_{x_1 \le r < x_2} \dot{r} &= \dot{r}\big|_{r=\sqrt{\frac{2x_1}{\pi\alpha}}} \\ &= \frac{\varepsilon}{\pi}\left(1 - \sqrt{2\pi\alpha x_1}\right). \end{aligned} \tag{S20}$$

We further divide the case by the sign of the right-hand-side of Eq. (S20).

(ii-a) If $\alpha \ge \frac{1}{2\pi x_1}$, then $\max\limits_{x_1 \le r < x_2} \dot{r} \le 0$ follows, which implies that $r = 0$ is the only fixed point of Eq. (14). Recall that $\dot{r}$ is a monotonically decreasing function for the intervals $r < x_1$ and $r \ge x_2$.

(ii-b) If $\alpha < \frac{1}{2\pi x_1}$, then $\max\limits_{x_1 \le r < x_2} \dot{r} > 0$ follows. According to the inequality (S19), this case occurs only when

$$\frac{1}{2\pi x_1} > \frac{2x_1}{\pi x_2^2} \iff x_2 > 2x_1. \tag{S21}$$

In this case, new fixed points (one is stable and the other is unstable) of Eq. (14) appear by the saddle-node bifurcation, whose coordinates are given by

$$r_{\text{stable}} = \begin{cases} \frac{1+\sqrt{1-2\alpha\pi x_1}}{\alpha\pi} & \text{if} \quad \alpha \ge \frac{2(x_2-x_1)}{\pi x_2^2}, & \text{(S22a)} \\ \sqrt{\frac{2(x_2-x_1)}{\alpha\pi}} & \text{if} \quad \alpha < \frac{2(x_2-x_1)}{\pi x_2^2}, & \text{(S22b)} \end{cases}$$

and

$$r_{\text{unstable}} = \frac{1 - \sqrt{1 - 2\alpha\pi x_1}}{\alpha\pi}, \tag{S23}$$

respectively. Note that the right-hand-side of Eqs. (S22a) and (S23) are the roots of the right-hand-side of Eq. (14b), and the right-hand-side of Eq. (S22b) is the positive root of the right-hand-side of Eq. (14c). We also mention that Eq. (S22) is divided by the sign of

$$\dot{r}\big|_{r=x_2} = -\frac{\varepsilon x_2}{2}\left[\alpha - \frac{2(x_2 - x_1)}{\pi x_2^2}\right]. \tag{S24}$$

(iii) Finally, we consider the case where $\sqrt{\frac{2x_1}{\pi\alpha}} \geq x_2$, which is equivalent to

$$\alpha \leq \frac{2x_1}{\pi x_2^2}. \tag{S25}$$

In this case, $\dot{r}$ is a monotonically increasing function for the interval $x_1 \leq r < x_2$. Then, we see that Eq. (14) has the nontrivial two fixed points, one of which is stable and the other is unstable, if and only if

$$\dot{r}|_{r=x_2} > 0 \iff \alpha < \frac{2(x_2 - x_1)}{\pi x_2^2}. \tag{S26}$$

We also find that the coordinates of the stable and unstable fixed points are given as Eq. (S22b) and Eq. (S23), respectively. Note that if the inequality (S21) holds, then the inequality (S25) satisfies the inequality (S26) (i.e., $\frac{2x_1}{\pi x_2^2} < \frac{2(x_2-x_1)}{\pi x_2^2}$ follows if $x_2 > 2x_1$).

Based on the above discussion, we clarify the dynamics of Eq. (14), which are summarized in the main article.

5 The case where g is given by rational function with a linear numerator and quadratic denominator

Here, we describe another case where g is given by the following smooth rational function to model the escapement mechanism:

$$g(x, y) := \begin{cases} \frac{x}{1+x^2} & \text{if} \quad xy > 0, \\ 0 & \text{otherwise.} \end{cases} \tag{S27}$$

By substituting Eq. (8) into Eq. (S27), we obtain

$$g(r\cos\phi, -r\sin\phi) = \begin{cases} \dfrac{r\cos\phi}{1 + r^2\cos^2\phi} & \text{if} \quad (2n + \frac{1}{2})\pi < \phi < (2n+1)\pi \\ & \quad \text{or} \quad (2n - \frac{1}{2})\pi < \phi < 2n\pi, \\ 0 & \text{otherwise,} \end{cases} \tag{S28}$$

with arbitrary integer n. Then, it follows from Eqs. (12) and (S28) that

$$\begin{aligned} \bar{g}_1(r) &= \frac{1}{2\pi}\left(\int_{\frac{\pi}{2}}^{\pi} \frac{r\cos\phi\sin\phi d\phi}{1 + r^2\cos^2\phi} + \int_{\frac{3}{2}\pi}^{2\pi} \frac{r\cos\phi\sin\phi d\phi}{1 + r^2\cos^2\phi}\right) \\ &= \frac{-1}{4\pi r}\left\{\left[\log(1 + r^2\cos^2\phi)\right]_{\frac{\pi}{2}}^{\pi} + \left[\log(1 + r^2\cos^2\phi)\right]_{\frac{3}{2}\pi}^{2\pi}\right\} \\ &= \frac{-\log(1 + r^2)}{2\pi r}, \end{aligned} \tag{S29}$$

and

$$\begin{aligned} \bar{g}_2(r) &= \frac{1}{2\pi}\left(\int_{\frac{\pi}{2}}^{\pi} \frac{r\cos^2\phi d\phi}{1 + r^2\cos^2\phi} + \int_{\frac{3}{2}\pi}^{2\pi} \frac{r\cos^2\phi d\phi}{1 + r^2\cos^2\phi}\right) \\ &= \frac{r}{\pi}\int_{-\infty}^{0} \frac{du}{(1 + u^2)(1 + u^2 + r^2)} \qquad (u := \tan\phi) \\ &= \frac{1}{\pi r}\int_{-\infty}^{0}\left(\frac{1}{1 + u^2} - \frac{1}{1 + u^2 + r^2}\right)du \\ &= \frac{1}{\pi r}\left[\text{Arctan}\, u - \frac{1}{\sqrt{1 + r^2}}\text{Arctan}\left(\frac{u}{\sqrt{1 + r^2}}\right)\right]_{-\infty}^{0} \\ &= \frac{1}{2r}\left(1 - \frac{1}{\sqrt{1 + r^2}}\right). \end{aligned} \tag{S30}$$

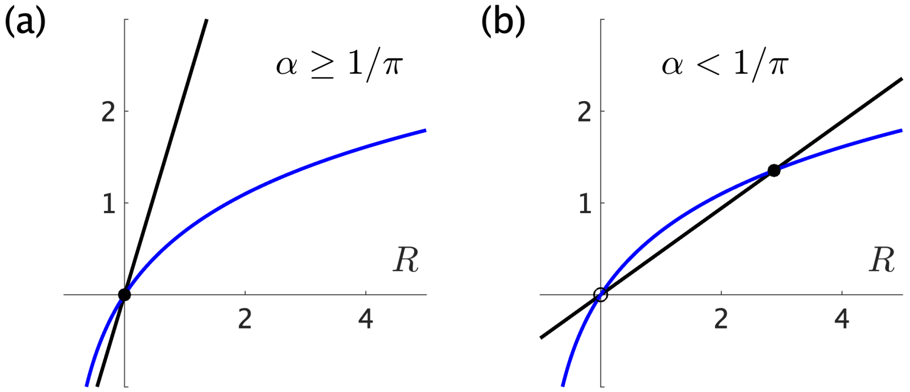

Figure S1: This figure shows the solutions of the transcendental equation (S32). The black and blue lines represent $\pi\alpha R$ and $\log(1+R)$, respectively. Both the black dot and black circle show the solutions of Eq. (S32). Note that the black dot corresponds to the stable fixed point of Eq. (S31a), while the black circle corresponds to the unstable fixed point. (**a**) If α equals to or is larger than $1/\pi$, which is the value when the black line is tangent to the blue curve, Eq. (S32) has the unique solution $R = 0$. We set $\alpha = 0.7$ to depict this panel. (**b**) If $\alpha < 1/\pi$, Eq. (S32) has another solution other than $R = 0$. We set $\alpha = 0.15$ to depict this panel.

In the derivation of Eq. (S30), we use the relationships $\cos^2\phi = \frac{1}{1+u^2}$ and $d\phi = \frac{du}{1+u^2}$. According to Eqs. (S29) and (S30), we see that averaged equations (i.e., Eq. (11)) are calculated as

$$\dot{r} = -\frac{\varepsilon\alpha}{2} r + \frac{\varepsilon}{2\pi r}\log(1+r^2), \tag{S31a}$$

$$r\dot{\theta} = -\frac{\varepsilon}{2r}\left(1 - \frac{1}{\sqrt{1+r^2}}\right). \tag{S31b}$$

We consider the dynamics of Eq. (S31a). Obviously, the fixed point of Eq. (S31a) satisfies the following transcendental equation:

$$\pi\alpha R = \log(1+R), \tag{S32}$$

where $R \coloneqq r^2 \geq 0$. Since $\log(1+R)$ is a concave function of R, we see that Eq. (S32) has the unique solution ($R = 0$) if $\alpha \geq 1/\pi$ and two solutions if $\alpha < 1/\pi$ (Fig. S1).

Thus, as there exists a one-to-one relationship between $r \geq 0$ and R, we find the following:

- If $\alpha > 1/\pi$, Eq. (S31a) has a stable fixed point $r = 0$.
- The transcritical bifurcation occurs at $\alpha = 1/\pi$.
- If $\alpha < 1/\pi$, Eq. (S31a) has an unstable fixed point ($r = 0$) and a stable fixed point, whose value is the positive solution of Eq. (S32).

Figures 5 (**a**) and (**b**) show the typical flows of Eq. (S31a) before and after the bifurcation point. We see that, as α decreases, the trivial fixed point $r = 0$ changes to unstable and non-trivial fixed point emerges due to the transcritical bifurcation. The bifurcation diagram for r is shown in Fig. 5 (**c**). The green cross marks show the numerically obtained equilibrium states of Eq. (6) when we increase α, which agree with the analytically obtained bifurcation diagram (black lines). However, in contrast to Model (i), (ii), and (iii) in the main article, this model is inappropriate to simulate the dynamics of metronome because it cannot simulate the bistability of the resting state and the oscillatory state.

We consider that this problem arises because the escapement mechanism expressed as Eq. (S27) is not sufficiently weak near the resting state. In other words, Eq. (S27) implies that $g(x, y) \simeq x$ in the vicinity of $x = 0$. These are the reasons why we use in the main article the rational function with numerator of degree 3 and denominator of degree 4 such that $g(x, y) \simeq 0$ holds in the vicinity of $x = 0$ by a first-order approximation.

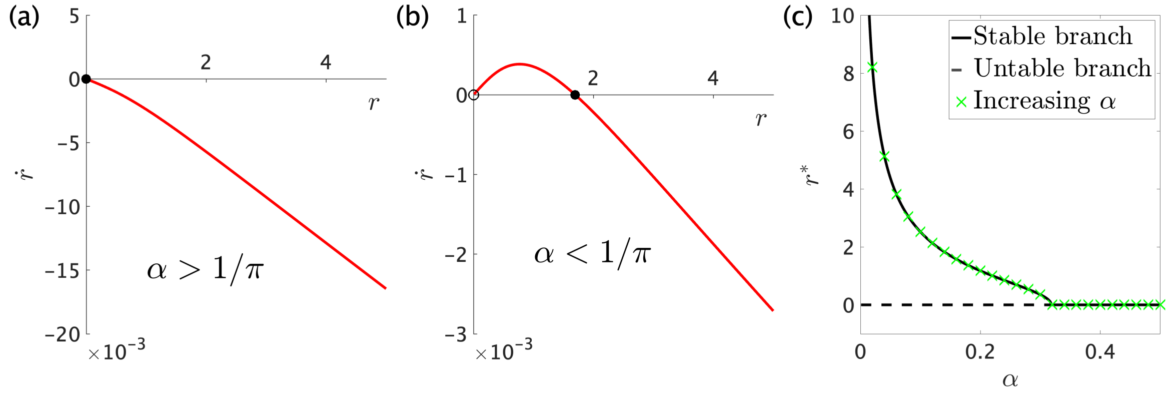

Figure S2: (**a, b**) The typical flows of Eq. (S31a), in which we plot $\dot{r}$ as a function of r. The black dot and black circle in each panel represent the stable and unstable fixed points, respectively. As for the parameter values, we set $\varepsilon = 0.01$, $\alpha = 0.7$ in panel (**a**), and $\alpha = 0.15$ in panel (**b**). (**c**) The bifurcation diagram for r obtained by both the averaging approximation and the numerical simulation of Eq. (6) where g is given by Eq. (S27). The solid and dashed lines represent the analytically obtained stable fixed point (the positive solution of Eq. (S32) if $\alpha < 1/\pi$ and $r^* = 0$ if $\alpha \geq 1/\pi$) and the unstable fixed point ($r^* = 0$ if $\alpha < \pi$), respectively. The green cross marks show the equilibrium solutions obtained by numerical simulation of Eq. (6). We first use $\alpha = 0.02$ and then increase α by 0.02 until α reaches to 0.5. The initial condition for the first simulation is $x(0) = 2.0, \dot{x}(0) = 0$. For the following simulations, we use the equilibrium state of the previous simulation as the initial condition. We fix $\varepsilon = 0.01$ for all the simulations in panel (**c**).

6 Calculation of $\bar{g}_{1,2}(r)$ in the case where g is given as Eq. (19)

By substituting Eq. (8) into Eq. (19), we obtain

$$g(r\cos\phi, -r\sin\phi) = \begin{cases} \dfrac{r^3\cos^3\phi}{1+r^4\cos^4\phi} & \text{if} \quad (2n+\frac{1}{2})\pi < \phi < (2n+1)\pi \\ & \quad \text{or} \quad (2n-\frac{1}{2})\pi < \phi < 2n\pi, \\ 0 & \text{otherwise}, \end{cases} \tag{S33}$$

with arbitrary integer n. Then, it follows from Eqs. (12) and (S33) that

$$\begin{aligned} \bar{g}_1(r) &= \frac{1}{2\pi}\left(\int_{\frac{\pi}{2}}^{\pi} \frac{r^3\cos^3\phi\sin\phi d\phi}{1+r^4\cos^4\phi} + \int_{\frac{3}{2}\pi}^{2\pi} \frac{r^3\cos^3\phi\sin\phi d\phi}{1+r^4\cos^4\phi}\right) \\ &= \frac{-1}{8\pi r}\left\{\left[\log(1+r^4\cos^4\phi)\right]_{\frac{\pi}{2}}^{\pi} + \left[\log(1+r^4\cos^4\phi)\right]_{\frac{3}{2}\pi}^{2\pi}\right\} \\ &= \frac{-\log(1+r^4)}{4\pi r}, \end{aligned} \tag{S34}$$

and

$$
\begin{aligned}
\bar{g}_2(r) &= \frac{1}{2\pi}\left(\int_{\frac{\pi}{2}}^{\pi}\frac{r^3\cos^4\phi d\phi}{1+r^4\cos^4\phi}+\int_{\frac{3}{2}\pi}^{2\pi}\frac{r^3\cos^4\phi d\phi}{1+r^4\cos^4\phi}\right)\\
&= \frac{r^3}{\pi}\int_{-\infty}^{0}\frac{du}{(1+u^2)\left[(1+u^2)^2+r^4\right]} \qquad (u:=\tan\phi)\\
&= \frac{1}{\pi r}\int_{-\infty}^{0}\left(\frac{1}{1+u^2}-\frac{1+u^2}{(1+u^2)^2+r^4}\right)du\\
&= \frac{1}{\pi r}\left[\operatorname{Arctan}u\right]_{-\infty}^{0}-\frac{1}{\pi r}\int_{-\infty}^{0}\left(\frac{-\frac{\sqrt{\sqrt{1+r^4}-1}}{2\sqrt{2(1+r^4)}}u+\frac{1}{2\sqrt{1+r^4}}}{u^2+\sqrt{2(\sqrt{1+r^4}-1)}u+\sqrt{1+r^4}}+\frac{\frac{\sqrt{\sqrt{1+r^4}-1}}{2\sqrt{2(1+r^4)}}u+\frac{1}{2\sqrt{1+r^4}}}{u^2-\sqrt{2(\sqrt{1+r^4}-1)}u+\sqrt{1+r^4}}\right)du\\
&= \frac{1}{2r}-\frac{1}{\pi r}\left[-\frac{\sqrt{\sqrt{1+r^4}-1}}{4\sqrt{2(1+r^4)}}\log\left(u^2+\sqrt{2(\sqrt{1+r^4}-1)}u+\sqrt{1+r^4}\right)\right.\\
&\quad+\frac{\sqrt{2(\sqrt{1+r^4}+1)}}{4\sqrt{1+r^4}}\operatorname{Arctan}\left(\sqrt{\frac{2}{1+\sqrt{1+r^4}}}\left(u+\sqrt{\frac{\sqrt{1+r^4}-1}{2}}\right)\right)\\
&\quad+\frac{\sqrt{\sqrt{1+r^4}-1}}{4\sqrt{2(1+r^4)}}\log\left(u^2-\sqrt{2(\sqrt{1+r^4}-1)}u+\sqrt{1+r^4}\right)\\
&\quad\left.+\frac{\sqrt{2(\sqrt{1+r^4}+1)}}{4\sqrt{1+r^4}}\operatorname{Arctan}\left(\sqrt{\frac{2}{1+\sqrt{1+r^4}}}\left(u-\sqrt{\frac{\sqrt{1+r^4}-1}{2}}\right)\right)\right]_{-\infty}^{0}\\
&= \frac{1}{2r}-\frac{\sqrt{2(\sqrt{1+r^4}+1)}}{4r\sqrt{1+r^4}}+\frac{\sqrt{\sqrt{1+r^4}-1}}{4\sqrt{2(1+r^4)}}\lim_{u\to-\infty}\log\left(\frac{u^2-\sqrt{2(\sqrt{1+r^4}-1)}u+\sqrt{1+r^4}}{u^2+\sqrt{2(\sqrt{1+r^4}-1)}u+\sqrt{1+r^4}}\right)\\
&= \frac{1}{2r}\left(1-\sqrt{\frac{1+\sqrt{1+r^4}}{2(1+r^4)}}\right). \qquad \text{(S35)}
\end{aligned}
$$

According to Eqs. (S34) and (S35), we see that Eq. (11) are calculated as Eqs. (20a) and (20b).

7 Calculation of $\bar{g}_{1,2}(r)$ in the case where g is given as Eq. (24)

By substituting Eq. (8) into Eq. (24), we obtain

$$
g(r\cos\phi,-r\sin\phi)=\begin{cases} ar^3\cos^3\phi-br^5\cos^5\phi & \text{if}\quad (2n+\frac{1}{2})\pi<\phi<(2n+1)\pi \\ & \quad\text{or}\quad (2n-\frac{1}{2})\pi<\phi<2n\pi, \\ 0 & \text{otherwise,}\end{cases} \qquad \text{(S36)}
$$

with arbitrary integer n. Then, it follows from Eqs. (12) and (S36) that

$$
\begin{aligned}
\bar{g}_1(r) &= \frac{1}{2\pi}\left[\int_{\frac{\pi}{2}}^{\pi}(ar^3\cos^3\phi-br^5\cos^5\phi)\sin\phi d\phi\right.\\
&\quad\left.+\int_{\frac{3}{2}\pi}^{2\pi}(ar^3\cos^3\phi-br^5\cos^5\phi)\sin\phi d\phi\right]\\
&= \frac{-1}{\pi}\left[\frac{ar^3\cos^4\phi}{4}-\frac{br^5\cos^6\phi}{6}\right]_{\frac{\pi}{2}}^{\pi}\\
&= \frac{-(3ar^3-2br^5)}{12\pi}, \qquad \text{(S37)}
\end{aligned}
$$

and

$$\bar{g}_2(r) = \frac{1}{2\pi}\left[\int_{\frac{\pi}{2}}^{\pi}(ar^3\cos^4\phi - br^5\cos^6\phi)d\phi + \int_{\frac{3}{2}\pi}^{2\pi}(ar^3\cos^4\phi - br^5\cos^6\phi)d\phi\right]$$
$$= \frac{1}{\pi}\left[ar^3\left(\frac{\sin 4\phi}{32} + \frac{\sin 2\phi}{4} + \frac{3\phi}{8}\right) - br^5\left(\frac{\sin 6\phi}{192} + \frac{3\sin 4\phi}{64} + \frac{15\sin 2\phi}{64} + \frac{5\phi}{16}\right)\right]_{\frac{\pi}{2}}^{\pi}$$
$$= \frac{6ar^3 - 5br^5}{32}. \tag{S38}$$

According to Eqs. (S37) and (S38), we see that Eq. (11) are calculated as Eqs. (25a) and (25b).

8 Derivation of Eq. (42)

We introduce the complex variables $A_i(t)$ that satisfy

$$x_i(t) = \frac{1}{2}(A_i(t)e^{it} + c.c.), \tag{S39a}$$
$$y_i(t) = \frac{1}{2}(iA_i(t)e^{it} + c.c.). \tag{S39b}$$

Noting that $A_i = (x_i - iy_i)e^{-it}$, we transform Eq. (39) into

$$\dot{A}_i = ie^{-it}\varepsilon\left[\mu(x_1 + x_2) + \beta y_i - g(x_i, y_i)\right]. \tag{S40}$$

Equations (S40) are derived in the same way as the one-oscillator system, which is described in Sec. 1 in Supplementary Information.

We express $A_i(t)$ in a polar coordinate as below:

$$A_i(t) = r_i(t)e^{i\theta_i(t)}, \tag{S41}$$

where $r_i(t), \theta_i(t)$ are real variables with $r_i(t) \geq 0$. Then, Eq. (40) follows from Eqs. (S39) and (S41). By substituting Eqs. (S41) and (40) into Eq. (S40), we have

$$\dot{r}_i + ir_i\dot{\theta}_i = ie^{-i\phi_i}\varepsilon\left[\mu(r_1\cos\phi_1 + r_2\cos\phi_2) - \beta r_i\sin\phi_i - g(r_i\cos\phi_i, -r_i\sin\phi_i)\right], \tag{S42}$$

from which we obtain Eq. (42).

9 Stability analysis of two-oscillator system

We perform the linear stability analysis for the fixed points $(r_1, r_2, \psi) = (r^*, r^*, 0)$ and (r^*, r^*, π) under the condition (50). The Jacobian matrix J at these fixed points can be written in a unified manner as below:

$$J = \begin{pmatrix} j_1 & 0 & -j_2 \\ 0 & j_1 & j_2 \\ j_3 & -j_3 & 0 \end{pmatrix}, \tag{S43}$$

where

$$j_1 := \frac{\varepsilon}{12\pi}\left(-6\pi\beta + 9ar^{*2} - 10br^{*4}\right), \tag{S44}$$
$$j_2 := \frac{\varepsilon\mu r^* s}{2}, \tag{S45}$$
$$j_3 := \frac{\varepsilon}{8}\left(3ar^* - 5br^{*3} + \frac{8\mu s}{r^*}\right), \tag{S46}$$

and

$$s := \begin{cases} 1 & \text{if} \quad \psi = 0, \\ -1 & \text{if} \quad \psi = \pi. \end{cases} \tag{S47}$$

The eigenvalues of J are

$$j_1, \quad \frac{j_1 \pm \sqrt{j_1^2 - 8 j_2 j_3}}{2}, \tag{S48}$$

which implies that each of the fixed points (47) and (48) is asymptotically stable if and only if

$$j_1 < 0 \quad \text{and} \quad j_2 j_3 > 0. \tag{S49}$$

Below, we examine the sign of j_1, j_2, and j_3.

Obviously, $j_2 > 0$ if $\psi = 0$ and $j_2 < 0$ if $\psi = \pi$. By substituting Eq. (49) into Eqs. (S44) and (S46), we get

$$j_1 = -\frac{\varepsilon}{8\pi b}\left(3a^2 - 16\pi b\beta + a\sqrt{9a^2 - 48\pi b\beta}\right), \tag{S50}$$

$$j_3 = -\frac{\varepsilon(27a^2 - 120\pi b\beta + 9a\sqrt{9a^2 - 48\pi b\beta} - 64 b\mu s)}{32\sqrt{(3a + \sqrt{9a^2 - 48\pi b\beta})b}}. \tag{S51}$$

It thus follows from inequality (50) and Eqs. (S50) and (S51) that

$$j_1 < 0, \tag{S52}$$

and

$$j_3 < 0 \quad \text{if} \quad \psi = \pi. \tag{S53}$$

If $\psi = 0$ (i.e., if $s = 1$), then $j_3 > 0$ is true when inequality (51) holds.

Based on the above discussion, we conclude that the fixed point $(r^*, r^*, 0)$ is always asymptotically stable and that the fixed point (r^*, r^*, π) is asymptotically stable under the condition (51).

10 Supplementary figures

We present several figures that support the main article.

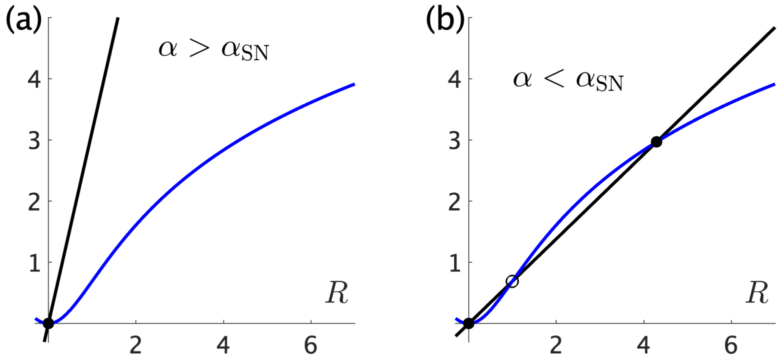

Figure S3: This figure shows the solutions of the transcendental equation (21). The black and blue lines represent $2\pi\alpha R$ and $\log(1 + R^2)$, respectively. Both the black dot and black circle show the solutions of Eq. (21). Note that the black dot corresponds to the stable fixed point of Eq. (20a), while the black circle corresponds to the unstable fixed point. (**a**) If α is larger than $\alpha_{\rm SN}$, which is the value when the black line is tangent to the blue curve, Eq. (21) has the unique solution $R = 0$. We set $\alpha = 0.5$ to depict this panel. (**b**) If $\alpha < \alpha_{\rm SN}$, Eq. (21) has two solutions in addition to $R = 0$. We set $\alpha = 0.11$ to depict this panel.

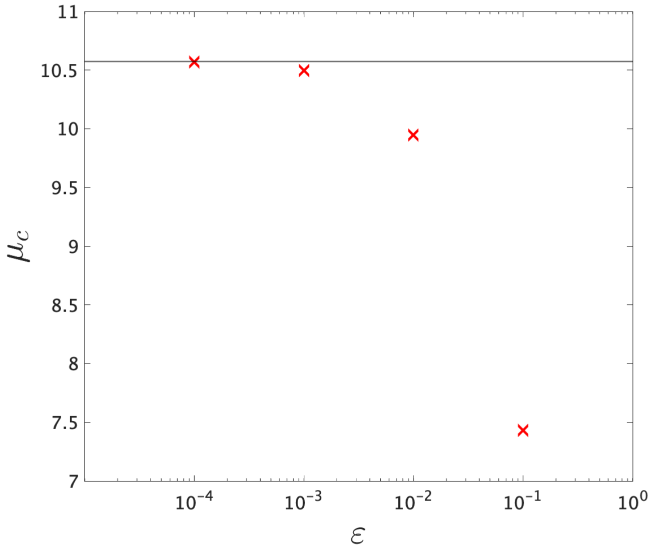

Figure S4: The relashionship between ε and $\mu_{\rm c}$, the value of μ at which the stability of the in-phase synchronization switches. The red cross marks show numerically obtained $\mu_{\rm c}$, derived by numerical integration of Eq. (37) and bisection method. The black line shows $\mu_{\rm c}$ in inequality (51), which is obtained by averaging approximation. Note that the red cross marks approach the black line as we decrease ε. We fix $a = 4, b = 1, \beta = 0.3$ in this figure.

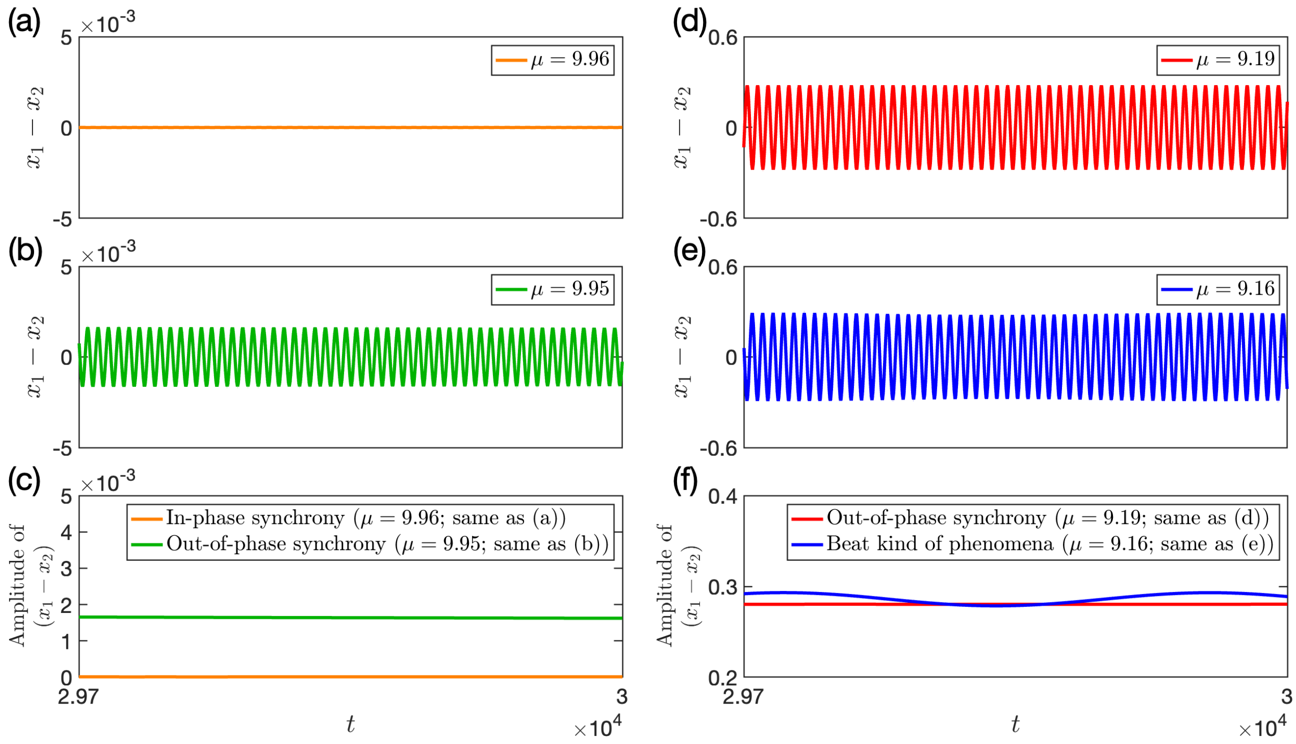

Figure S5: Panels (**a-c**) and (**d-f**) illustrate the origin and bifurcation of the out-of-phase synchrony (Fig. 5 (**c**)) and the beat-like dynamics (Fig. 5 (**b**)), respectively. We fix $\beta = 0.3$ for the whole figure. (**a**) When $\mu = 9.96$, the in-phase synchronization is stable. (**b**) When we decrease μ, the in-phase synchronization is no longer stable and the value $x_1(t) - x_2(t)$ begins to oscillate, which corresponds to the out-of-phase synchronization seen in Fig. 5 (**c**). (**c**) We re-plot the dynamics seen in panels (**a**) and (**b**) changing the y-axis to the amplitude of $(x_1(t) - x_2(t))$. The emergence of a stable fixed point near the unstable one suggests that the out-of-phase synchronization occurs by the supercritical pitchfork bifurcation. (**d**) When $\mu = 9.19$, the out-of-phase synchronization is stable. (**e**) When we decrease μ, the out-of-phase synchronization becomes unstable. The amplitude of $x_1(t) - x_2(t)$ begins to oscillate, which corresponds to the beat kind of solution seen in Fig. 5 (**b**). (**f**) The time course of the amplitude of $(x_1(t) - x_2(t))$. We see that the beat-like dynamics occur by the supercritical Hopf bifurcation. The initial condition for panel (**e**) is the slightly perturbed state from the equilibrium solution of panel (**d**) (i.e., $(x_1(0), y_1(0), x_2(0), y_2(0)) = (x_1^\dagger + 0.01, y_1^\dagger, x_2^\dagger, y_2^\dagger)$ where $(x_1^\dagger, y_1^\dagger, x_2^\dagger, y_2^\dagger)$ is the equilibrium solution in panel (**d**)). For the rest of the simulation, we use initial condition 1 described in the main article.
